# Discovery of potential urine-accessible metabolite biomarkers associated with muscle disease and corticosteroid response in the *mdx* mouse model for Duchenne

**DOI:** 10.1371/journal.pone.0219507

**Published:** 2019-07-16

**Authors:** Mathula Thangarajh, Aiping Zhang, Kirandeep Gill, Habtom W. Ressom, Zhenzhi Li, Rency S. Varghese, Eric P. Hoffman, Kanneboyina Nagaraju, Yetrib Hathout, Simina M. Boca

**Affiliations:** 1 Department of Neurology, George Washington University School of Medicine and Children’s National Health Systems, Washington, D.C., United States of America; 2 Department of Genomics and Precision Medicine, George Washington University School of Medicine and Children’s National Health Systems, Washington, D.C., United States of America; 3 Department of Oncology, Georgetown University Medical Center, Washington, D.C., United States of America; 4 School of Pharmacy & Pharmaceutical Sciences, Binghamton University, Binghamton, N.Y., United States of America; 5 Innovation Center for Biomedical Informatics, Georgetown University Medical Center, Washington, D.C., United States of America; 6 Department of Biostatistics, Bioinformatics and Biomathematics, Georgetown University Medical Center, Washington, D.C., United States of America; Swinburne University of Technology, AUSTRALIA

## Abstract

Urine is increasingly being considered as a source of biomarker development in Duchenne Muscular Dystrophy (DMD), a severe, life-limiting disorder that affects approximately 1 in 4500 boys. In this study, we considered the *mdx* mice—a murine model of DMD—to discover biomarkers of disease, as well as pharmacodynamic biomarkers responsive to prednisolone, a corticosteroid commonly used to treat DMD. Longitudinal urine samples were analyzed from male age-matched *mdx* and wild-type mice randomized to prednisolone or vehicle control via liquid chromatography tandem mass spectrometry. A large number of metabolites (869 out of 6,334) were found to be significantly different between *mdx* and wild-type mice at baseline (Bonferroni-adjusted p-value < 0.05), thus being associated with disease status. These included a metabolite with m/z = 357 and creatine, which were also reported in a previous human study looking at serum. Novel observations in this study included peaks identified as biliverdin and hypusine. These four metabolites were significantly higher at baseline in the urine of *mdx* mice compared to wild-type, and significantly changed their levels over time after baseline. Creatine and biliverdin levels were also different between treated and control groups, but for creatine this may have been driven by an imbalance at baseline. In conclusion, our study reports a number of biomarkers, both known and novel, which may be related to either the mechanisms of muscle injury in DMD or prednisolone treatment.

## Introduction

Oral corticosteroids are the standard of care in Duchenne Muscular Dystrophy (DMD)—an X-linked genetic disease that affects 1 in 4000 to 5000 boys worldwide [1, 2]. DMD is caused by mutations in the dystrophin gene located on the short arm of the X chromosome [3, 4]. Oral corticosteroid use in young boys with DMD changes the natural history of the disease. One of the largest natural history studies in DMD showed that corticosteroids increased survival in DMD, improved motor outcomes such as continued ambulation, as well as cardiopulmonary outcomes [5]. Despite these unambiguous clinical benefits, there is great variation in the dosage and regimen of corticosteroids, and with compliance to treatment in DMD [6]. Patients and families often discontinue corticosteroids mainly due to unpleasant side-effects including sleep disturbance, weight gain, increased fracture risk, and metabolic syndrome. Identification of non-invasive pharmacodynamic biomarkers associated with glucocorticoid side effects in boys may help predict those individuals who might be at a higher risk for developing corticosteroid-induced side-effects [7].

We have previously identified a metabolic signature consisting of serum biomarkers in DMD [8]. Metabolites that were increased in the serum of DMD patients compared to age-matched healthy controls included creatine, arginine, and unknown compounds with m/z values of 357.25 and 312.01 Da; metabolites that were decreased in the serum of DMD patients compared to age-matched healthy controls included creatinine, androgen derivatives, and other yet-to-be-identified metabolites. To further develop metabolite biomarkers for DMD, we used the *mdx* mouse—a widely used pre-clinical model—to agnostically evaluate urine metabolites associated with dystrophin deficiency and/or response to corticosteroids. Urine is an easily accessible bodily fluid, can be collected non-invasively and on multiple time-points, and is becoming an attractive source of biomarker development in DMD [9–15]. The identification of a palette of pharmacodynamic urine metabolites will allow the identification of metabolic pathways that are affected by the disease pathogenesis and that might be responsive to therapies such as corticosteroids and exon skipping. Such pharmacodynamic biomarkers may also help future drug development and dose finding.

## Methods

### Animals

The experiments using mice described in this paper were conducted according to the institutional guidelines regarding the humane treatment of animals. This study was done in compliance with the National Institutes of Health guidelines for pre-clinical studies as described by [16] and the standard operating procedure for pre-clinical studies [17]. The protocol was approved by the Institutional Animal Care and Use Committee (IACUC) at Children’s National Health System (#30424).

Age-matched 5-week-old male wild-type (C57BL/10ScSnJ) and *mdx* (C57BL/10ScSn-*Dmd*<*mdx*>/J) mice litters were purchased from Jackson Laboratory (Bar Harbor, ME). Mice were acclimatized for 4 days prior to commencement of experiments. A total of 48 mice (23 wild-type (WT) and 25 *mdx*) were used to ensure a rigorous statistical analysis. WT and *mdx* mice matched for body weight were randomized into treatment with either vehicle control (cherry syrup) or prednisolone (dissolved in cherry syrup) for a total of 4 weeks as previously published [18]. Animals were treated with vehicle or prednisolone (5mg/kg/day) by mouth based on body weight. Animals had access to water and food ad libitum. The study team was blinded to the treatment groups. Unblinding of the different treatment groups occurred after data collection and data processing.

### Urine collection

**Fig 1:**
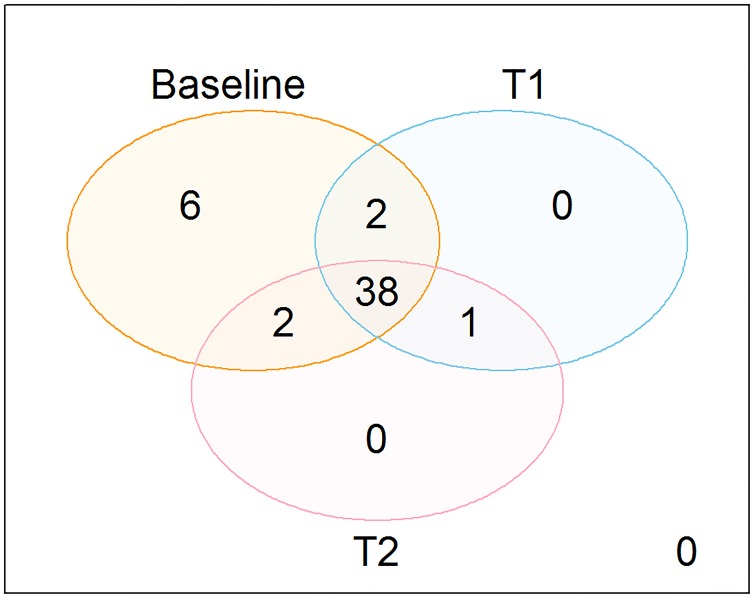
Overlaps between the number of mice at baseline, T1, and T2.

Urine from mice was collected by placing them in clean cages early in the morning. Urine was collected at three different time points: baseline (prior to treatment), 15 days following commencement of treatment, and at completion of treatment at 4 weeks; these are coded as times T0, T1, and T2 in the remainder of this manuscript. We note that at T0, the mice had not received any vehicle cherry syrup. The urine collection had the following caveats: 4 *mdx* and 2 WT mice died between times T0 and T1, and some mice did not urinate at every time point; one sample was not available for T2 and therefore not analyzed. In total, 48 mice were evaluated at time T0 and 43 unique mice at times T1 and T2, out of which 41 provided measurements at T1 but not T2 and 41 provided measurements at T2 but not T1. The number of mice categorized by genotype, time point, and treatment is provided in Table 1. Fig 1 provides the overlap between the 3 time points in terms of the number of mice.

**Table 1:** The number of mice in each category, split by genotype, time point, and treatment. Note that no treatment was given at baseline.

		Time				
		Baseline	T1		T2	
			Prednisolone	Vehicle control	Prednisolone	Vehicle control
Genotype	*mdx*	25	11	9	10	10
	WT	23	11	10	11	10
	Subtotal by treatment		22	19	21	20
	Total by time point	48	41		41	

### LC-MS method

For metabolite extraction, 80 *μ*l of a solution of 50% acetonitrile in water containing internal standards (10*μ*l of 1mg/ml debrisoquine and 50*μ*l of 1mg/ml 4- nitrobenzoic acid added to 10 ml of 50% acetonitrile in water) was added to 20 *μ*l of each urine sample in an Eppendorf vial. The samples were centrifuged at 13,000 rpm for 20 minutes at 4°C and the supernatant transferred to fresh vials for UPLC-Qtof analysis. A 2*μ*l aliquot from each sample was injected onto a Waters Acquity BEH C18 1.7 *μ*m, 2.1 × 50 mm column using an Acquity UPLC system by Waters Corporation, Milford, MA connected to a quadrupole time of flight mass spectrometer (Waters G2-Qtof). The total run time for each injection was 11 minutes. Metabolites were eluted from the column at a flow rate of 0.5 mL/min using a gradient mobile phase consisting of solvent A—100% water with 0.1% formic acid—and solvent B—100% acetonitrile with 0.1% formic acid. The gradient started at 5% of solvent B and gradually ramped to 20% of solvent B in 4 minutes at curve 6. Then it ramped to 95% of solvent B in 4 minutes and maintained at 95% of solvent B for an additional 9 minutes to wash the column before returning to initial conditions of 95% solvent A and 5% solvent B in 2 minutes. Mass spectrometry analysis was performed by electrospray ionization in both positive and negative mode at a capillary voltage of 3.0 kV and sampling cone voltage of 30 V. The source temperature was set to 120°C and the desolvation temperature to 500°C. The cone gas flow was maintained at 25L/hr and desolvation gas flow at 1000L/hr. Leucine-encephalin solution in 50% acetonitrile was used as internal calibrant in positive mode with [M+H]^+^ = 556.2771 and negative mode with [M-H]^−^ = 554.2615, with an overall mass error of ± 10 ppm. The data were acquired in centroid mode from mass range of 50 to 1200 with the software MassLynx (Waters Corporation). Pooled quality controls (QC) samples were injected after every 10 injections. Two QC samples were also injected at the beginning, leading to 15 QC samples in total.

### Data processing

LC-MS metabolomics samples were preprocessed as previously described [8]. In brief, the XCMS approach [19], available through the *xcms* package on Bioconductor [20] was used to detect features and estimate intensities, perform retention time correction, and group peaks from different samples, followed by filling in missing peaks via integrating the signal in the peak region defined by the other samples. The resulting peaks were then annotated using the *CAMERA* package, [21] resulting in the prediction of possible isotopes.

Following these initial preprocessing steps, peak groups with fewer than 37 peaks were removed, along with predicted isotopes. This led to 3,674 peaks in the positive mode and 2,663 peaks in the negative mode. Internal standard normalization was then performed, with the intensities in each mode being divided by the corresponding standard and the standards being removed from the list of peaks, leading to a total number of 6,335 peaks. A very small number of peak-sample combinations had missing values, coded as intensities of 0 (51 for the negative mode and 3 for the positive mode); these intensities were replaced by the smallest non-zero intensity for that peak across all samples. The intensities were then log2-transformed and the peaks from both modes were quantile-normalized together [22]. These data are in S1 File.

### Principal components analysis (PCA)

Prior to individual metabolite-level statistical analyses, we performed a principal components analysis (PCA) using the peaks detected in both modes and all the urine samples, including the QC samples. Thus, each principal component corresponds to a linear combination of the normalized, log2-transformed metabolite measurements. We considered this approach to better understand the variation in our dataset and identify possible outliers or artifacts, as recommended in [23]. All the QC samples were removed from the downstream analyses following the PCA.

### Statistical analyses

Given that the mice had a different diet at time T0 compared to times T1 and T2, the analyses considered were: 1) a comparison of the genotypes at time T0 via a two-sample t-test and 2) a mixed effects linear model for times T1-T2 which included genotype, time, and treatment, as well as all their interactions, with a random intercept to account for the within-mouse correlation. We note that for analysis 1) we did not consider treatment given that all the mice were at baseline, with none being yet treated with prednisolone. Prior to analysis 1), a single peak that had the same value in all samples at T0 was removed, so that this analysis was performed on 6,334 peaks. Levene’s test was initially applied to each peak to test whether the variances were equal within the groups defined by genotypes. The percent of null hypotheses, i.e. of peaks that have equal variances was estimated via the *qvalue* package [24, 25] as 82%. We also compared the p-values from two-sample t-tests assuming equal variances and the two-sample t-tests that do not make that assumption (Welch’s t-test.) Given that the vast majority of peaks appear to have equal variance between genotype groups and that the results between the two methods are very similar (correlation between p-values > 0.9999), moving forward, we used the results from the analysis with the equal variance assumption. For analysis 2), we focused on the top 50 most significant peaks from analysis 1), with the addition of creatine and creatinine, which are well-known to be important in DMD and were among the top metabolites associated with disease status in previous work [8]. In the mixed effects models, likelihood ratio tests were used to assess the association of genotype, time, and treatment with the transformed peak intensities. To account for multiple testing, statistical significance was determined to be reached if the Bonferroni-adjusted p-values were less than 0.05, when considering all 6,334 peaks, corresponding to a critical p-value of 7.89 × 10^−6^. All analyses were performed using the *R* statistical programming language. [26] Our code is available at https://github.com/SiminaB/DMD-mdx-pred-metabolomics.

For analysis 2), we used the lme function in the *nlme* package in R [27] to fit linear mixed effects models, using the maximum likelihood approach. The outcome is the normalized, log2-transformed metabolite intensity. For each of the 52 metabolites considered, a “full” model is fit considering genotype, time, and treatment and all the genotype x time x treatment interactions as fixed effects, with a random intercept fit for each mouse, to account for the within-mouse correlation:

$$\begin{array}{ccc} Y_{it} & = & \beta_{0}+\beta_{1}1\left({\text{Genotype}}_{i}=mdx\right)+\beta_{2}1\left({\text{Time}}_{it}=T_{2}\right)+\\ & & \beta_{3}1\left({\text{Treatment}}_{i}=\text{Prednisolone}\right)+\\ & & \beta_{4}1\left({\text{Genotype}}_{i}=mdx\right)1\left({\text{Time}}_{it}=T_{2}\right)+\\ & & \beta_{5}1\left({\text{Genotype}}_{i}=mdx\right)1\left({\text{Treatment}}_{i}=\text{Prednisolone}\right)+\\ & & \beta_{6}1\left({\text{Genotype}}_{i}=mdx\right)1\left({\text{Time}}_{it}=T_{2}\right)1\left({\text{Treatment}}_{i}=\text{Prednisolone}\right)+\\ & & u_{i}+\epsilon_{it}, \end{array}$$

where *i* indicates the mouse, *t* indicates 1 of 2 measurements per mouse, *u*_*i*_ indicates the mouse-specific random intercept, and *ϵ*_*it*_ indicates the random noise term. We note that *u*_*i*_ and *ϵ*_*it*_ are both random variables with independent normal distributions and their own variances, which can be denoted by $\sigma_{u}^{2}$ and $\sigma_{\epsilon }^{2}$.

“Null” models are then fit that exclude genotype (includes time, treatment, time x treatment), time (includes genotype, treatment, genotype x treatment), treatment (includes genotype, time, genotype x time). Thus, each null model is nested in the full model. To obtain the association of each metabolite with genotype, the full model is compared to the model that excludes genotype using the *anova* function in R, which compares the likelihoods of the fitted linear mixed models and reports a p-value from the likelihood ratio test.

### LC-MS/MS experiments for identification of top peaks

The top 50 peaks in terms of significant differences between *mdx* and WT mice at baseline were considered as queries in the Human Metabolome Database (HMDB), looking for possible H+, Na+, and K+ adducts for the positive mode peaks and H- adducts for the negative mode peaks, within 10 ppm mass error. Eleven of these peaks were selected for identification by MS/MS. Most of them were also significantly different between the genotypes longitudinally. Creatine and creatinine were previously identified, so we did not include them in the MS/MS experiments here. The positive mode peaks considered in the MS/MS experiment had the following m/z values: 229.16, 234.18, 357.25, 485.33, 583.26, 705.18, 884.93 Da. The negative mode peaks considered in the MS/MS experiment had the following m/z values: 210.11, 486.16, and 652.11 Da. The MS/MS experiments were similar to those in [8], running the samples on Waters G2-Qtof. Pooled QC samples were processed similarly to the profiling experiment and the chromatographic conditions used for data acquisition remained the same. In particular, checks were performed by measuring the accuracy of the metmix (cocktail of standards) peaks which fall under 10 ppm. The metmix is run before the start of the batch and after the end of the batch. The MS/MS spectra obtained were then inspected by matching to the online available spectra from the databases Metlin [28] and HMDB [29] and manually. Further putative identification was performed as follows: The MZXML files were parsed using the *pyteomics* python library [30]. For each sample, the retention time was used to access the scan of the targeted compound. The mzxml function in *pyteomics* was used to access the m/z and corresponding intensity information of the retention time. The top 30 peaks with highest intensities were used for compound identification using MS/MS spectral libraries such as Metlin, HMDB, and MONA (http://mona.fiehnlab.ucdavis.edu/). In addition to searching tools provided by these sources, we used MetaboQuest (http://omicscraft.com/MetaboQuest) for spectral matching considering +/- 10 ppm mass tolerance for both precursor and fragment peaks.

## Results

### Principal components analysis (PCA)

**Fig 2:**
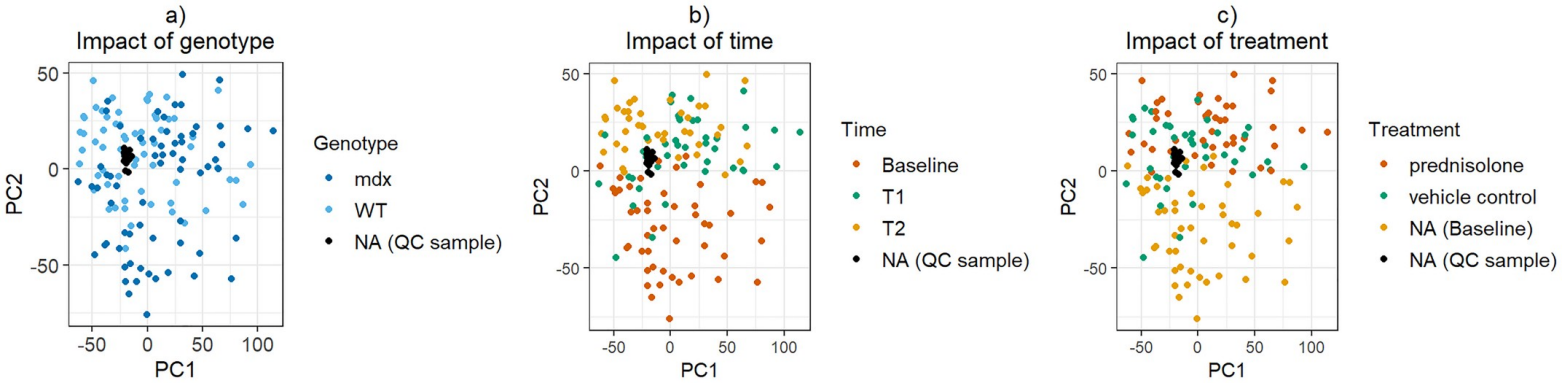
PCA plots of the internal standard normalized, quantile normalized, and log2-transformed intensity data. PC2 is plotted against PC1. Each circle corresponds to an individual sample. In panel a), samples are color-coded by genotype, in panel b), by time point, and in panel c), by treatment.

The PCA analysis showed that 23% of the variability can be explained by the first principal component (PC1) and 12% by the second principal component (PC2). Each individual remaining principal component explained less than 10% of the remaining variability. Fig 2 shows plots of PC2 against PC1. We note that there appear to be no outlying samples and that all the QC samples cluster together, a good indication of data quality. There are no disjoint clusters based on either genotype (Panel a), time (Panel b), or treatment (panel c), which is a positive indication of the absence of batch effects. However, the baseline samples are somewhat separated from the T1 and T2 samples in PC2, strengthening our decision to consider them in a separate analysis.

### Comparison of genotypes at baseline

Out of a total of 6,334 detected peaks, 868 were determined to show significant differences between the *mdx* and the WT mice (Bonferroni-adjusted p-value < 0.05). Given this large number, for the downstream analyses, we chose to focus on the top 50 most significant peaks, along with creatine (m/z = 132.08) and creatinine (m/z = 114.06), as described above. Results for these peaks are in S2 File, including the m/z values, the mode, the retention time in seconds, the p-value, and the log2 fold change (difference in means between the mdx and WT groups after normalization, log2 transformation). The results from the HMDB query for the top peaks with a window of 10 ppm are also in S2 File. 11 of these 50 peaks were selected for additional annotation and identification via MS/MS. The combined annotation approach using the HMDB+Metlin+MONA+MetaboQuest search yielded one additional annotation for the peak at 234.18, namely 5-hydroxyiminoisocaryophyllene, within a 10 ppm window. The MS/MS spectra for the 11 selected peaks considered in this follow-up experiment are in S1 Fig.

**Fig 3:**
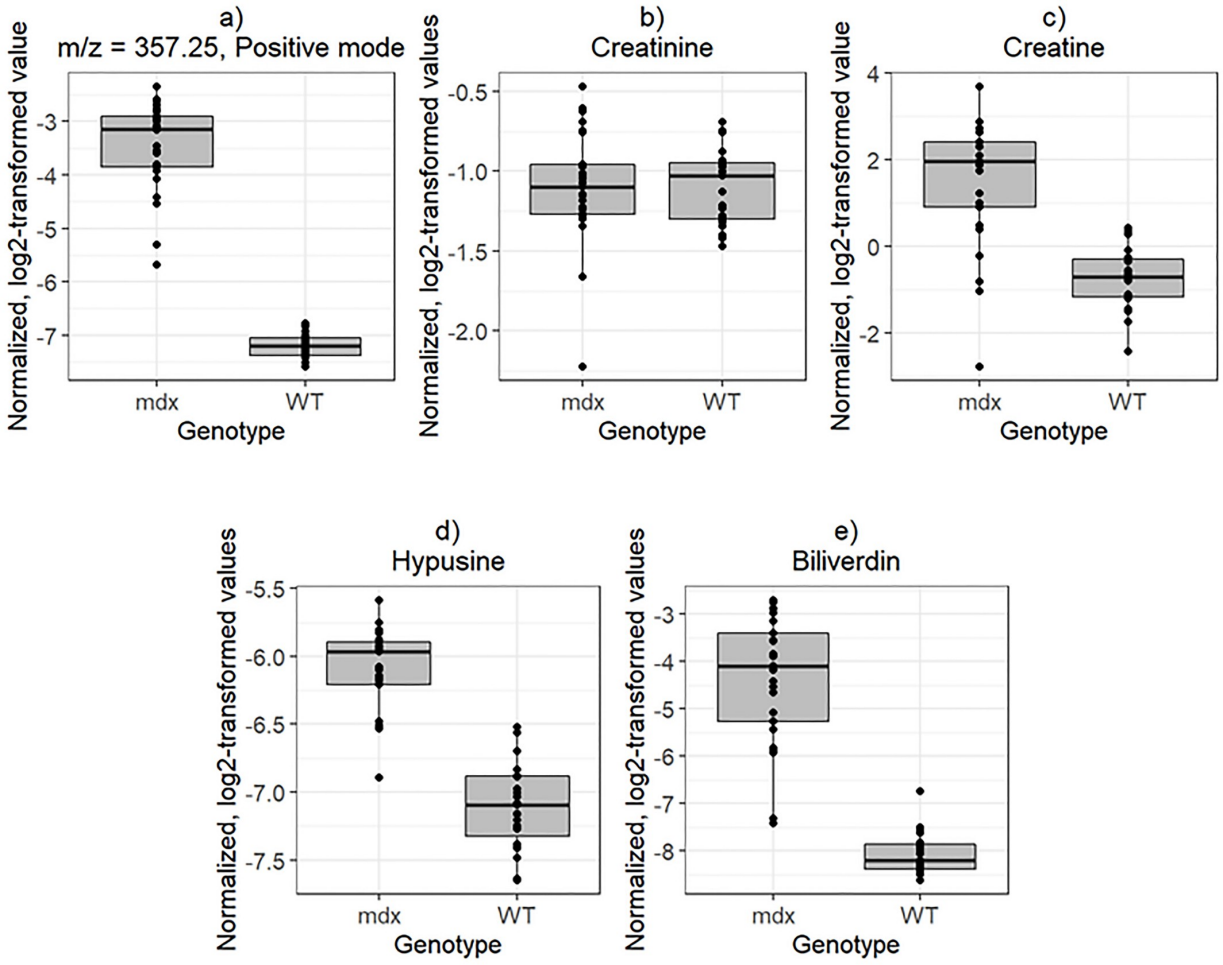
Genotype comparison of normalized, log2-transformed intensity values at baseline (T0). a) peak with m/z = 357.25, b) creatine (m/z = 132.08 in positive mode), c) creatinine (m/z = 114.06 in positive mode), d) hypusine (m/z = 234.18 in positive mode), e) biliverdin (m/z = 583.26 in positive mode).

Creatine and creatinine are known to be important in DMD and were among the top peaks in our human serum study [8]. We note that the positive mode peak, with m/z = 357.25 (p = 5.11 x 10^-25^ unadjusted), appears to be the same as the top peak in our previous work with DMD patients [8]. Interestingly, creatine was not among the top 50 peaks in the mouse model, but ranked 472 and was still deemed significant (p = 4.91 x 10^-8^ unadjusted). Creatinine, however, was not significant (p = 0.98 unadjusted). Boxplots representing results for these 3 peaks for the comparison between genotype are shown in Fig 3a–3c. We considered two additional peaks at m/z = 234.18 and m/z = 583.26 which were significant (p = 1.26 x 10^-14^ unadjusted and p = 2.31 x 10^-16^ unadjusted, respectively) and identified using MS/MS in Fig 3d and 3e. The peak at m/z = 234.18 was identified as hypusine and the peak at m/z = 583.26 was identified as a geometric isomer of biliverdin in accordance with recently published MS/MS data of biliverdin [31]. For all peaks in Fig 3 besides creatinine, the intensities are significantly elevated in the urine of *mdx* mice compared to WT mice. In particular, for the peak with m/z = 357.25, this finding recapitulates our earlier findings from the serum of younger individuals with DMD, assuming the two peaks represent the same metabolite. For creatine, older boys with DMD had higher values [8].

### Comparison of genotypes, time, and treatment for T1-T2

As discussed above, we focused our analysis at the later time points on the peaks found to have the lowest 50 p-values in the genotype comparison at T0, in addition to creatine and creatinine. This is because we are of the opinion that it is important to focus on peaks that are likely to be indicative of the disease process, with differences seen at an early age. Creatine and all the peaks selected for the MS/MS experiments except for the one at m/z = 370.04 were among those associated with genotype at T1-T2. Creatine, biliverdin, and the peaks at m/z = 210.11, 484.16, 884.93, and 485.33 were significantly associated with treatment at T1-T2. Creatine and all the peaks selected for the MS/MS experiments except for the one at m/z = 484.16 were among those associated with time. Full results for the T1-T2 analysis are found in S3 File.

**Fig 4:**
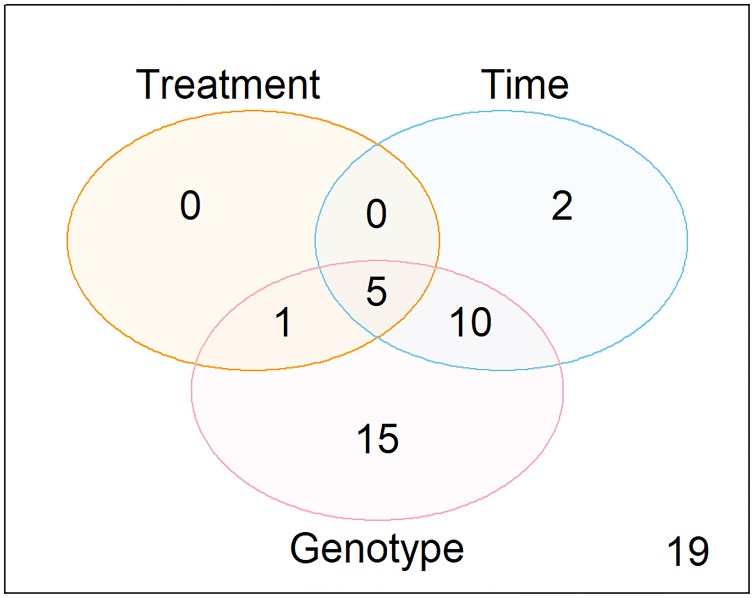
Overlaps between the peaks significantly associated with treatment, time, and genotype at times T1-T2. Significance was defined as Bonferroni-adjusted p-value < 0.05 for 6,334 peaks. The peaks considered at times T1-T2 were out of the 52 peaks considered, consisting of the top 50 peaks most significantly associated with genotype at T0, plus creatine and creatinine. 19 peaks were not significantly associated with treatment, time, or genotype (bottom right corner).

Overall, 31 of these 52 peaks were significantly associated with genotype, 6 with treatment, and 17 with time. The overlaps between these categories are summarized in Fig 4.

**Fig 5:**
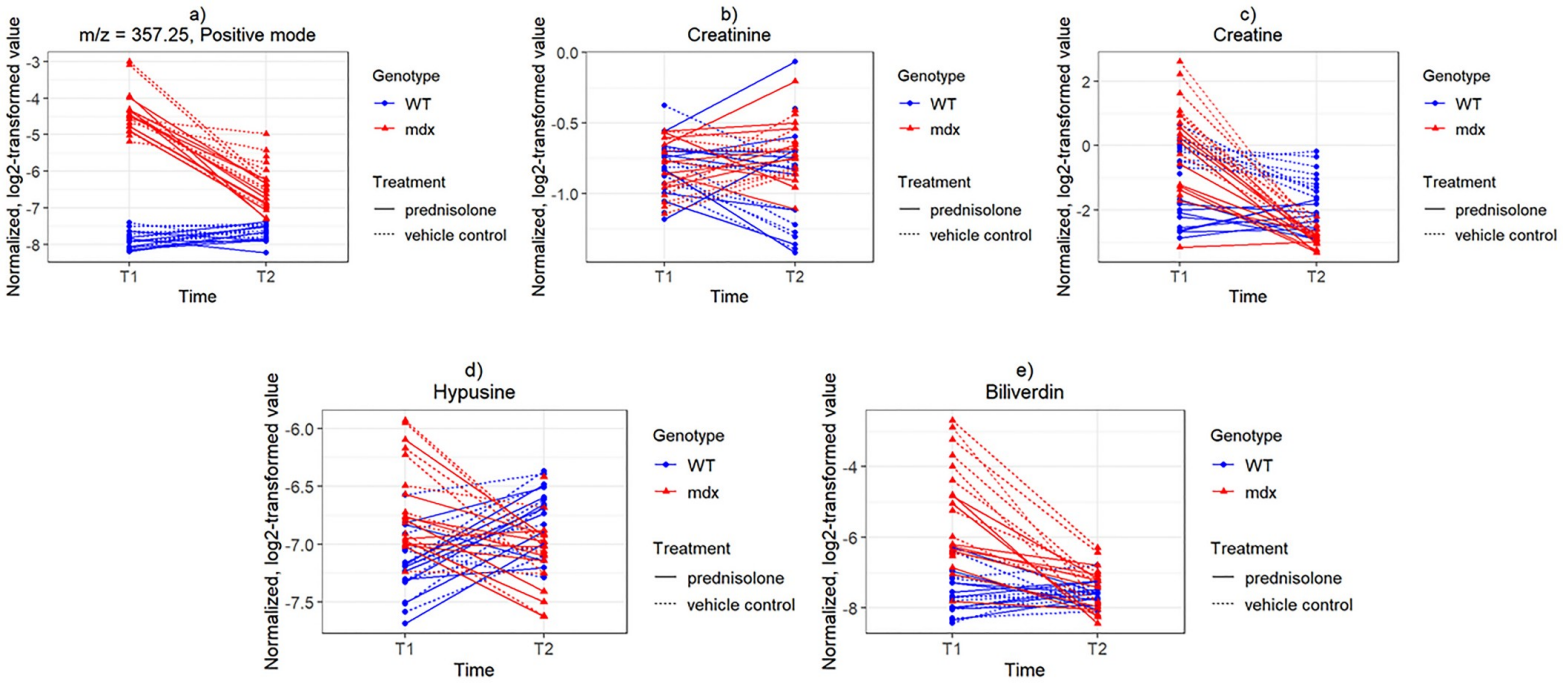
Normalized, log2-transformed intensity values at times T1-T2. a) peak with m/z = 357.25 in positive mode, b) creatine (m/z = 132.08 in positive mode), c) creatinine (m/z = 114.06 in positive mode), d) hypusine (m/z = 234.18 in positive mode), e) peak likely to be biliverdin (m/z = 583.26 in positive mode).

In particular, we note that the peak with m/z = 357.25 was associated with genotype (p = 1.71 x 10^-40^ unadjusted) and time (p = 1.99 x 10^-25^ unadjusted), but not with treatment (p = 7.11 x 10^-4^ unadjusted, p = 1 when adjusting for multiple comparisons). Creatine was associated with genotype, as well as with treatment and time. However, the treatment association for creatine may be driven at least in part by an imbalance at baseline between the WT mice later randomized to prednisolone versus those randomized to the control group (p = 0.009). Creatinine was not significantly associated with any variable. Fig 5 shows the trends for these five metabolites. Hypusine and biliverdin were also strongly associated with genotype (p = 9.28 x 10^-9^ unadjusted, p = 1.59 x 10^-16^ unadjusted, respectively). Hypusine was also associated with time (p = 2.66 x 10^-8^ unadjusted), but not with treatment (p = 0.16 unadjusted). Biliverdin was associated with both treatment (p = 2.24 x 10^-6^ unadjusted) and time (p = 2.01 x 10^-14^ unadjusted) and did not show a major imbalance at baseline between future treatment assignments (p = 0.670 in WT, p = 0.799 in *mdx*).

**Fig 6:**
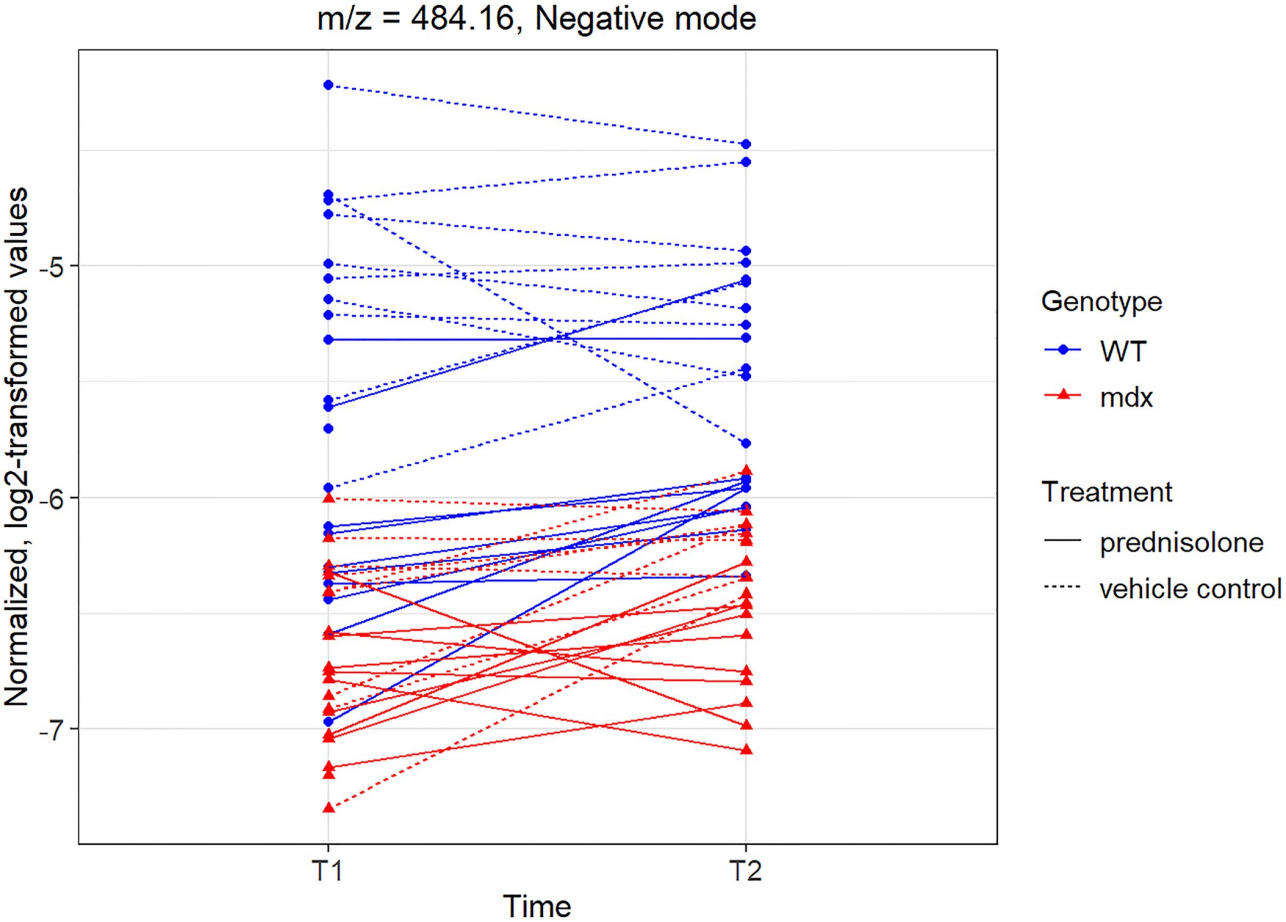
Normalized, log2-transformed intensity values at times T1-T2 for peak with m/z = 484.16 in negative mode.

Once again, the results for the peak with m/z = 357.25, showing a decrease over time in the genotype with the disorder, recapitulate the results from the human serum study [8]. An example of a peak showing treatment differences (p = 6.87 x 10^-9^ unadjusted) that did not have a large imbalance at baseline and that, interestingly, showed overall higher values in the *mdx* versus the WT group is the peak with m/z = 484.16, shown in Fig 6. S2 Fig shows the baseline (T0) results for this peak.

**Fig 7:**
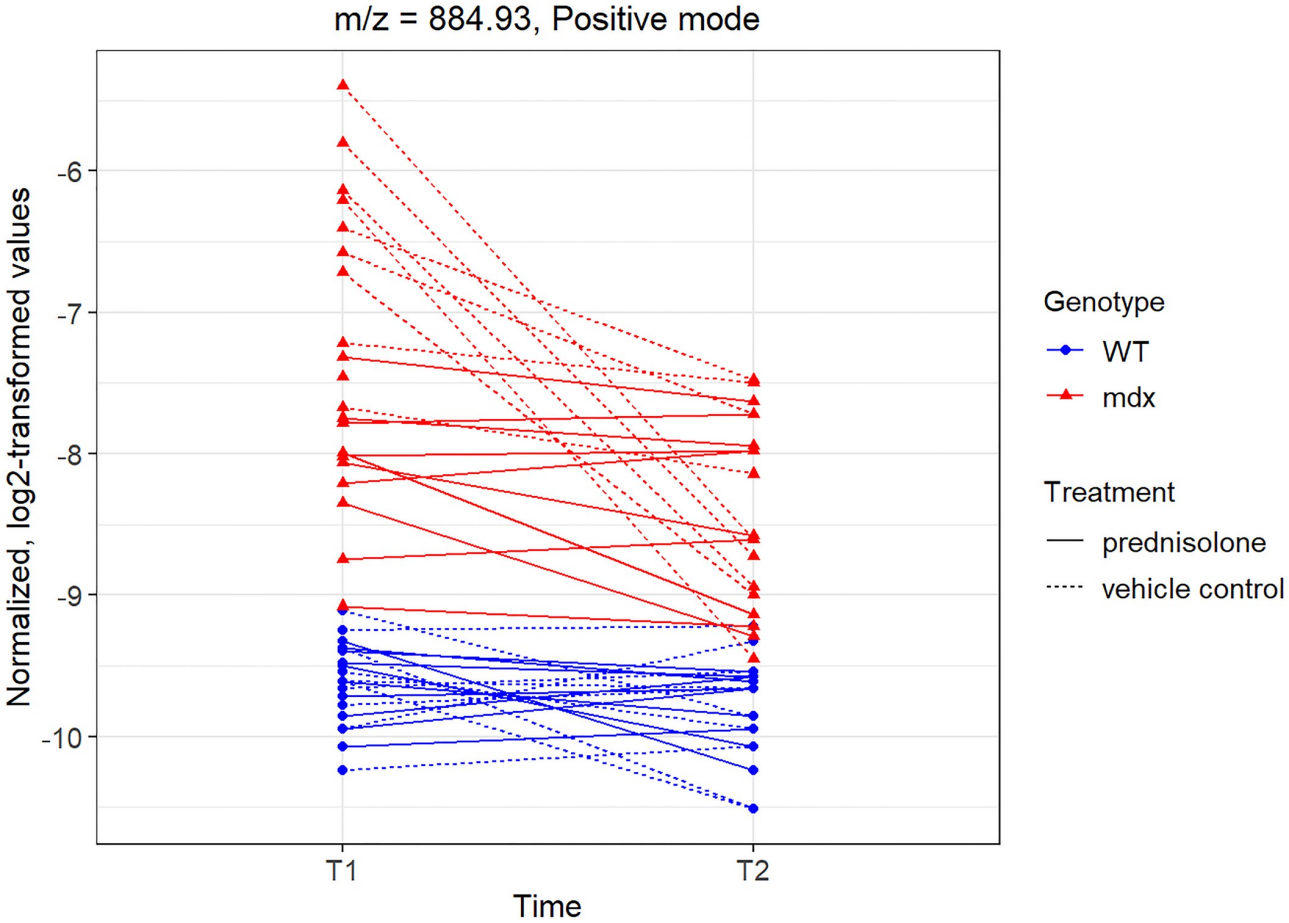
Normalized, log2-transformed intensity values at times T1-T2 for peak with m/z = 884.92 in positive mode.

We also highlight a peak with m/z = 884.92, which demonstrated a significant decrease over time, as well as a significant association with treatment (p = 1.37 x 10^-8^ unadjusted) and a clear separation between *mdx* and WT mice in Fig 7 (boxplot for time T0 in S3 Fig).

## Discussion

Our primary goal in this study was to identify urinary metabolite biomarkers associated with (i) muscle pathology and (ii) prednisone response in an animal model of DMD. We foresee that such information can guide future studies to evaluate biomarkers to predict disease progression and response to corticosteroids and other anti-inflammatory drugs. To our knowledge, this is the first study evaluating urine metabolites in the murine model of DMD. There is a growing body of literature that shows that urine is becoming an attractive biomarker in DMD [9–12] including that changes in urinary biomarker signatures may inform regarding response to therapy [32]. It is also easily accessible and less invasive, especially in very young boys and can be used to evaluate biomarker responses.

The myriad biomarkers reported here are reflective possibly of the different mechanisms that result in muscle injury in DMD. The best-known biomarkers of DMD is creatine kinase, which seeps from muscle into blood and is generally used to identify young boys with this disorder, being many times higher than in healthy individuals [33]. While this an appropriate diagnostic biomarker, we have shown that it decreases with disease progression and muscle loss [34]. We have also previously shown that creatine and creatinine are substantially different in the serum of DMD cases compared to controls, with creatine being higher in the cases and creatinine being lower; these metabolites are both in the creatine metabolism pathway, which includes creatine kinase [8]. Our current work shows an increase in creatine at baseline in the *mdx* mice over the WT mice, although it tends to decrease over time, whereas in the human serum study, it stayed constant or slightly increased over time in DMD cases and decreased in controls. However, creatinine was not significant in any of the analyses considered. Differences in these results could be due to the biospecimen (serum vs urine), model (human disease vs murine model), or type of study (observational natural history study vs randomized laboratory study). Recent work on urinary metabolites by a different group also found a significant increase of creatine —normalized to urinary creatinine —in mdx compared to WT mice at 3 months, though not at 1 month [15].

Particularly interesting is the metabolite corresponding to the peak with m/z = 357.25 (positive mode). This metabolite may have also been the top metabolite in our previous study [8]. Furthermore, it recapitulates the trend of being generally higher in cases than in controls, as well as decreasing over time in cases. While we have been unable to identify it herein, we consider that could be an important key to the pathogenesis of DMD and thus worthy of further investigation.

Two novel observations from our study concern the peaks identified as biliverdin and hypusine, metabolites which were higher in *mdx* urine compared to WT mice. At times T1 and T2, both metabolites showed associations with genotype and time, with biliverdin also showing an association with treatment. Biliverdin is an end-product in the heme pathway. Heme oxygenase catabolizes heme to equimolar biliverdin, carbon monoxide, and ferrous iron. Heme oxygenase is considered a regulator of oxidative stress and is anti-inflammatory. Second, its product carbon monoxide has vasorelaxant property. Recent studies have provided contradictory information on whether heme oxygenase-1 is decreased [35, 36] or increased [37, 38] in the muscle of *mdx* mice compared to controls. We postulate that a pro-inflammatory state seen in *mdx* mice drives active breakdown of heme, resulting in an increase in biliverdin levels in urine [39, 40]. Further investigation of this pathway could thus lead to both an improved understanding of the disease pathogenesis and a validated biomarker to monitor disease progression and response to treatment. Hypusine is an amino acid found exclusively in the eukaryotic translation factor 5A (eIF5A) family [41]. It is important in nonsense-mediated decay [42] but its role in muscle pathology has not yet been established. It is possible that the increase in hypusine is a reflection of ongoing extensive muscle regeneration in *mdx* mice, especially during early disease stages. It is also known that [43] eIF5A induces inflammatory cytokine cascade and the nitric oxide synthase iNOS and inhibition of eIF5A protects mice from developing diabetes. While the precise role of biliverdin and hypusine in vivo in skeletal muscle in *mdx* is unknown, we postulate that their levels in urine likely reflect inflammation and regeneration in dystrophin-deficient skeletal muscle.

The detection of a larger number of metabolite biomarkers in positive mode compared to negative mode was most likely driven by the low pH of the eluting solvent used in the LC-MS analysis (e.g. water and acetonitrile containing 1% formic acid), which favors protonation rather than deprotonation of metabolites. Additionally, some metabolites are more easily ionized in positive mode.

We note that our study has a number of important caveats. Most notably, we were unable to identify most of the 11 peaks selected for MS/MS, including the top peak with m/z = 357.25. We also found nearly 14% of peaks to be significantly different at baseline between the *mdx* and WT genotypes. While somewhat surprising, we focused only on the top 50 peaks with the most significant difference between *mdx* group and WT group in follow-up analyses and interpretation. Finding similar trends for the peak with m/z = 357.25 and for creatine as in our previous study is reassuring, as is the exploratory PCA analysis which showed the clustering of QC samples.

In conclusion, metabolome profiling of urine samples collected from *mdx* mice and WT mice enabled identification of a panel of potential metabolite biomarkers that might be associated with muscle pathogenesis. A subset of these metabolites was responsive to prednisolone treatment. These metabolites could prove to be useful biomarkers if evaluated in urine biospecimens of boys with DMD.

## Supporting information

S1 Fig Spectra for the 11 peaks selected for MS/MS identification. (PPTX)

S2 Fig Genotype comparison of normalized, log2-transformed intensity values at baseline (T0) for peak with m/z = 484.16 in negative mode. (TIF)

S3 Fig Genotype comparison of normalized, log2-transformed intensity values at baseline (T0) for peak with m/z = 884.92 in positive mode. (TIF)

S1 File Log2-transformed, quantile-normalized metabolite peak data. along with additional covariates. Metabolite peaks are labeled according to their m/z, mode, and retention time in seconds (RT). For the mouse samples considered in the statistical models: “Sample” column indicates mouse label and time point (T0, T1, or T2), separated by “_”; “SampleID” indicates mouse; “Time” indicates time point (T0, T1, or T2); “Genotype” is labeled “mdx23” or “WT”; “Group” is “group 1” (prednisolone) or “group 2” (vehicle control). The pooled quality control samples are labeled “QC” in the “Sample” and “SampleID” columns, “N” in the “Time” column, and NA in the “Genotype” and “Group” columns.
(TSV)

S2 File T-test and HMDB query results from HMDB query for top 50 peaks from baseline (T0) genotype analysis. A cutoff of 10 ppm was considered for the database search. The log2 fold change, corresponding to the mean difference between mdx and WT groups, the number of degrees of freedom of the test (DF), the 95% confidence interval (CI) lower and upper bounds, the t-statistic, and the p-value from the t-test are given, along with the rank of the peak and results from the HMDB query. Each row represents a possible HMDB annotation; if no metabolites are found within 10 ppm, the HMDB entries are labeled with “NA.”
(CSV)

S3 File Results from the linear mixed models for times T1 and T2, applied to the top 50 peaks from the baseline (T0) genotype analysis, along with creatine and creatinine. The first 8 sheets represent fixed effects (FE) estimates for the: intercept; genotype, time, and treatment main effects; genotype x time, genotype x treatment, and time x treatment pairwise interactions; and genotype x time x treatment interaction. They give the coefficient (Beta) estimate, its standard error (SE), the number of degrees of freedom (DF), the 95% confidence interval (CI) lower and upper bounds, the t-value, and the p-value. The next sheet presents the random effect (RE) estimates, which for these models are the random intercept standard deviations (SD). The last sheet presents the results from the likelihood ratio tests (LRT) comparing the full model with models that exclude genotype, time, and treatment, thus testing the associations with genotype, time, and treatment, respectively. The baseline values in the model were WT (for genotype), T1 (for time), and control (for treatment).
(XLSX)

## References

[1] Matthews E, Brassington R, Kuntzer T, Jichi F, Manzur AY. Corticosteroids for the treatment of Duchenne muscular dystrophy. The Cochrane Library. 2016; 10.1002/14651858.CD003725.pub4 PMC8580515 27149418

[2] Emery AE. Population frequencies of inherited neuromuscular diseases—a world survey. Neuromuscular disorders. 1991;1(1):19–29. 10.1016/0960-8966(91)90039-U 1822774

[3] Hoffman EP, Brown RH Jr, Kunkel LM. Dystrophin: the protein product of the Duchenne muscular dystrophy locus. Cell. 1987;51(6):919–928. 10.1016/0092-8674(87)90579-4 3319190

[4] Koenig M, Hoffman E, Bertelson C, Monaco A, Feener C, Kunkel L. Complete cloning of the Duchenne muscular dystrophy (DMD) cDNA and preliminary genomic organization of the DMD gene in normal and affected individuals. Cell. 1987;50(3):509–517. 10.1016/0092-8674(87)90504-6 3607877

[5] McDonald CM, Henricson EK, Abresch RT, Duong T, Joyce NC, Hu F, et al Long-term effects of glucocorticoids on function, quality of life, and survival in patients with Duchenne muscular dystrophy: a prospective cohort study. The Lancet. 2018;391(10119):451–461. 10.1016/S0140-6736(17)32160-8 29174484

[6] Griggs RC, Herr BE, Reha A, Elfring G, Atkinson L, Cwik V, et al Corticosteroids in Duchenne muscular dystrophy: major variations in practice. Muscle & nerve. 2013;48(1):27–31. 10.1002/mus.23831 23483575

[7] Hathout Y, Conklin LS, Seol H, Gordish-Dressman H, Brown KJ, Morgenroth LP, et al Serum pharmacodynamic biomarkers for chronic corticosteroid treatment of children. Scientific reports. 2016;6:31727 10.1038/srep31727 27530235 PMC4987691

[8] Boca SM, Nishida M, Harris M, Rao S, Cheema AK, Gill K, et al Discovery of metabolic biomarkers for Duchenne muscular dystrophy within a natural history study. PLOS ONE. 2016;11(4):e0153461 10.1371/journal.pone.0153461 27082433 PMC4833348

[9] Rouillon J, Zocevic A, Leger T, Garcia C, Camadro JM, Udd B, et al Proteomics profiling of urine reveals specific titin fragments as biomarkers of Duchenne muscular dystrophy. Neuromuscular Disorders. 2014;24(7):563–573. 10.1016/j.nmd.2014.03.012 24813925

[10] Takeshita E, Komaki H, Tachimori H, Miyoshi K, Yamamiya I, Shimizu-Motohashi Y, et al Urinary prostaglandin metabolites as Duchenne muscular dystrophy progression markers. Brain and Development. 2018;40(10):918–925. 10.1016/j.braindev.2018.06.012 30006121

[11] Rouillon J, Lefebvre T, Denard J, Puy V, Daher R, Ausseil J, et al High urinary ferritin reflects myoglobin iron evacuation in DMD patients. Neuromuscular Disorders. 2018;. 10.1016/j.nmd.2018.03.008 29776718

[12] Catapano F, Domingos J, Perry M, Ricotti V, Phillips L, Servais L, et al Downregulation of miRNA-29,-23 and-21 in urine of Duchenne muscular dystrophy patients. Epigenomics. 2018;(0). 10.2217/epi-2018-0022 29564913

[13] Lindsay A, Schmiechen A, Chamberlain CM, Ervasti JM, Lowe DA. Neopterin/7, 8-dihydroneopterin is elevated in Duchenne muscular dystrophy patients and protects mdx skeletal muscle function. Experimental Physiology. 2018;. 10.1113/EP087031 PMC6026059 29791760

[14] Lindsay A, McCourt PM, Karachunski P, Lowe DA, Ervasti JM. Xanthine oxidase is hyper-active in Duchenne muscular dystrophy. Free Radical Biology and Medicine. 2018;129:364–371. 10.1016/j.freeradbiomed.2018.10.404 30312761 PMC6599518

[15] Lindsay A, Chamberlain CM, Witthuhn BA, Lowe DA, Ervasti JM. Dystrophinopathy-associated dysfunction of Krebs cycle metabolism. Human Molecular Genetics. 2018;28(6):942–951. 10.1093/hmg/ddy404 PMC6400043 30476171

[16] Landis SC, Amara SG, Asadullah K, Austin CP, Blumenstein R, Bradley EW, et al A call for transparent reporting to optimize the predictive value of preclinical research. Nature. 2012;490(7419):187 10.1038/nature11556 23060188 PMC3511845

[17] Willmann R, De Luca A, Benatar M, Grounds M, Dubach J, Raymackers JM, et al Enhancing translation: guidelines for standard pre-clinical experiments in mdx mice. Neuromuscular Disorders. 2012;22(1):43–49. 10.1016/j.nmd.2011.04.012 21737275 PMC3227750

[18] Heier CR, Damsker JM, Yu Q, Dillingham BC, Huynh T, Van der Meulen JH, et al VBP15, a novel anti-inflammatory and membrane-stabilizer, improves muscular dystrophy without side effects. EMBO Molecular Medicine. 2013;5(10):1569–1585. 10.1002/emmm.201302621 24014378 PMC3799580

[19] Smith CA, Want EJ, O’Maille G, Abagyan R, Siuzdak G. XCMS: processing mass spectrometry data for metabolite profiling using nonlinear peak alignment, matching, and identification. Analytical Chemistry. 2006;78(3):779–787. 10.1021/ac051437y 16448051

[20] Gentleman RC, Carey VJ, Bates DM, Bolstad B, Dettling M, Dudoit S, et al Bioconductor: open software development for computational biology and bioinformatics. Genome Biology. 2004;5(10):R80 10.1186/gb-2004-5-10-r80 15461798 PMC545600

[21] Kuhl C, Tautenhahn R, Bottcher C, Larson TR, Neumann S. CAMERA: an integrated strategy for compound spectra extraction and annotation of liquid chromatography/mass spectrometry data sets. Analytical Chemistry. 2011;84(1):283–289. 10.1021/ac202450g 22111785 PMC3658281

[22] Bolstad BM, Irizarry RA, Åstrand M, Speed TP. A comparison of normalization methods for high density oligonucleotide array data based on variance and bias. Bioinformatics. 2003;19(2):185–193. 10.1093/bioinformatics/19.2.185 12538238

[23] Leek JT, Scharpf RB, Bravo HC, Simcha D, Langmead B, Johnson WE, et al Tackling the widespread and critical impact of batch effects in high-throughput data. Nature Reviews Genetics. 2010;11(10):733 10.1038/nrg2825 20838408 PMC3880143

[24] Storey JD. A direct approach to false discovery rates. Journal of the Royal Statistical Society: Series B (Statistical Methodology). 2002;64(3):479–498. 10.1111/1467-9868.00346

[25] Storey JD, with contributions from A J Bass, A Dabney, D Robinson. qvalue: Q-value estimation for false discovery rate control; 2015. Available from: http://github.com/jdstorey/qvalue.

[26] R Core Team. R: A Language and Environment for Statistical Computing; 2018 Available from: https://www.R-project.org/.

[27] Pinheiro J, Bates D, DebRoy S, Sarkar D, R Core Team. nlme: Linear and Nonlinear Mixed Effects Models; 2018 Available from: https://CRAN.R-project.org/package = nlme.

[28] Smith CA, O’Maille G, Want EJ, Qin C, Trauger SA, Brandon TR, et al METLIN: a metabolite mass spectral database. Therapeutic Drug Monitoring. 2005;27(6):747–751. 10.1097/01.ftd.0000179845.53213.39 16404815

[29] Wishart DS, Jewison T, Guo AC, Wilson M, Knox C, Liu Y, et al HMDB 3.0—the human metabolome database in 2013. Nucleic Acids Research. 2012;41(D1):D801–D807. 10.1093/nar/gks1065 23161693 PMC3531200

[30] Goloborodko AA, Levitsky LI, Ivanov MV, Gorshkov MV. Pyteomics—a Python framework for exploratory data analysis and rapid software prototyping in proteomics. Journal of the American Society for Mass Spectrometry. 2013;24(2):301–304. 10.1007/s13361-012-0516-6 23292976

[31] Frański R, Gierczyk B, Popenda Ł, Kasperkowiak M, Pędzinski T. Identification of a biliverdin geometric isomer by means of HPLC/ESI–MS and NMR spectroscopy. Differentiation of the isomers by using fragmentation “in-source”. Monatshefte für Chemie-Chemical Monthly. 2018;149(6):995–1002. 10.1007/s00706-018-2161-7 PMC5972171 29887645

[32] Antoury L, Hu N, Balaj L, Das S, Georghiou S, Darras B, et al Analysis of extracellular mRNA in human urine reveals splice variant biomarkers of muscular dystrophies. Nature Communications. 2018;9(1):3906 10.1038/s41467-018-06206-0 30254196 PMC6156576

[33] Okinaka S, Kumagai H, Ebashi S, Sugita H, Momoi H, Toyokura Y, et al Serum creatine phosphokinase: Activity in progressive muscular dystrophy and neuromuscular diseases. Archives of Neurology. 1961;4(5):520–525. 10.1001/archneur.1961.00450110050006 13730599

[34] Hathout Y, Brody E, Clemens PR, Cripe L, DeLisle RK, Furlong P, et al Large-scale serum protein biomarker discovery in Duchenne muscular dystrophy. Proceedings of the National Academy of Sciences. 2015;112(23):7153–7158. 10.1073/pnas.1507719112 PMC4466703 26039989

[35] Sun C, Yang C, Xue R, Li S, Zhang T, Pan L, et al Sulforaphane alleviates muscular dystrophy in mdx mice by activation of Nrf2. Journal of Applied Physiology. 2014;118(2):224–237. 10.1152/japplphysiol.00744.2014 25593219

[36] Chan MC, Ziegler O, Liu L, Rowe GC, Das S, Otterbein LE, et al Heme oxygenase and carbon monoxide protect from muscle dystrophy. Skeletal Muscle. 2016;6(1):41 10.1186/s13395-016-0114-6 27906108 PMC5126804

[37] Hnia K, Hugon G, Rivier F, Masmoudi A, Mercier J, Mornet D. Modulation of p38 mitogen-activated protein kinase cascade and metalloproteinase activity in diaphragm muscle in response to free radical scavenger administration in dystrophin-deficient Mdx mice. The American Journal of Pathology. 2007;170(2):633–643. 10.2353/ajpath.2007.060344 17255331 PMC1851881

[38] Pietraszek-Gremplewicz K, Kozakowska M, Bronisz-Budzynska I, Ciesla M, Mucha O, Podkalicka P, et al Heme oxygenase-1 influences satellite cells and progression of Duchenne muscular dystrophy in mice. Antioxidants & Redox Signaling. 2018;29(2):128–148. 10.1089/ars.2017.7435 29669436

[39] Ryter SW, Morse D, Choi AM. Carbon monoxide: to boldly go where NO has gone before. Science’s STKE. 2004;2004(230):re6–re6. 10.1126/stke.2302004re6 15114002

[40] Tongers J, Fiedler B, König D, Kempf T, Klein G, Heineke J, et al Heme oxygenase-1 inhibition of MAP kinases, calcineurin/NFAT signaling, and hypertrophy in cardiac myocytes. Cardiovascular Research. 2004;63(3):545–552. 10.1016/j.cardiores.2004.04.015 15276480

[41] Park MH. The post-translational synthesis of a polyamine-derived amino acid, hypusine, in the eukaryotic translation initiation factor 5A (eIF5A). The Journal of Biochemistry. 2006;139(2):161–169. 10.1093/jb/mvj034 16452303 PMC2494880

[42] Hoque M, Park JY, Chang Yj, Luchessi AD, Cambiaghi TD, Shamanna R, et al Regulation of gene expression by translation factor eIF5A: Hypusine-modified eIF5A enhances nonsense-mediated mRNA decay in human cells. Translation. 2017;5(2):e1366294 10.1080/21690731.2017.1366294 29034140 PMC5630042

[43] Maier B, Ogihara T, Trace AP, Tersey SA, Robbins RD, Chakrabarti SK, et al The unique hypusine modification of eIF5A promotes islet *β* cell inflammation and dysfunction in mice. The Journal of Clinical Investigation. 2010;120(6):2156–2170. 10.1172/JCI38924 20501948 PMC2877928

