# Supplementary material for: Discovery of potential urine-accessible metabolite biomarkers associated with muscle disease and corticosteroid response in the mdx mouse model for Duchenne
Source: PLoS One. 2019 Jul 16;14(7):e0219507. doi: 10.1371/journal.pone.0219507 (PMC6634414; doi:10.1371/journal.pone.0219507)
Supplement: S1 Fig — (PPTX) [file pone.0219507.s001.pptx]

## Slide 1
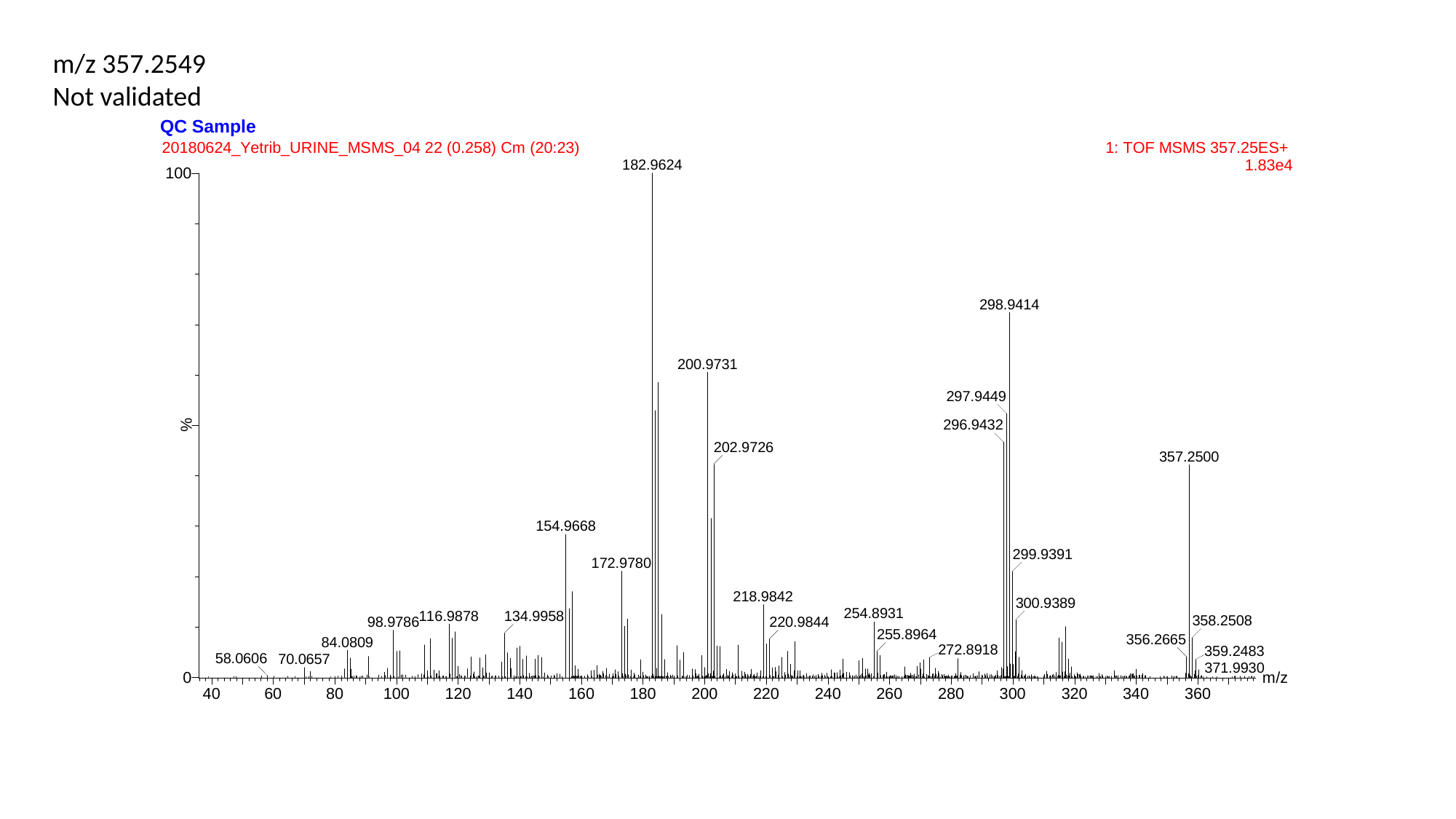

m/z 357.2549
Not validated

## Slide 2
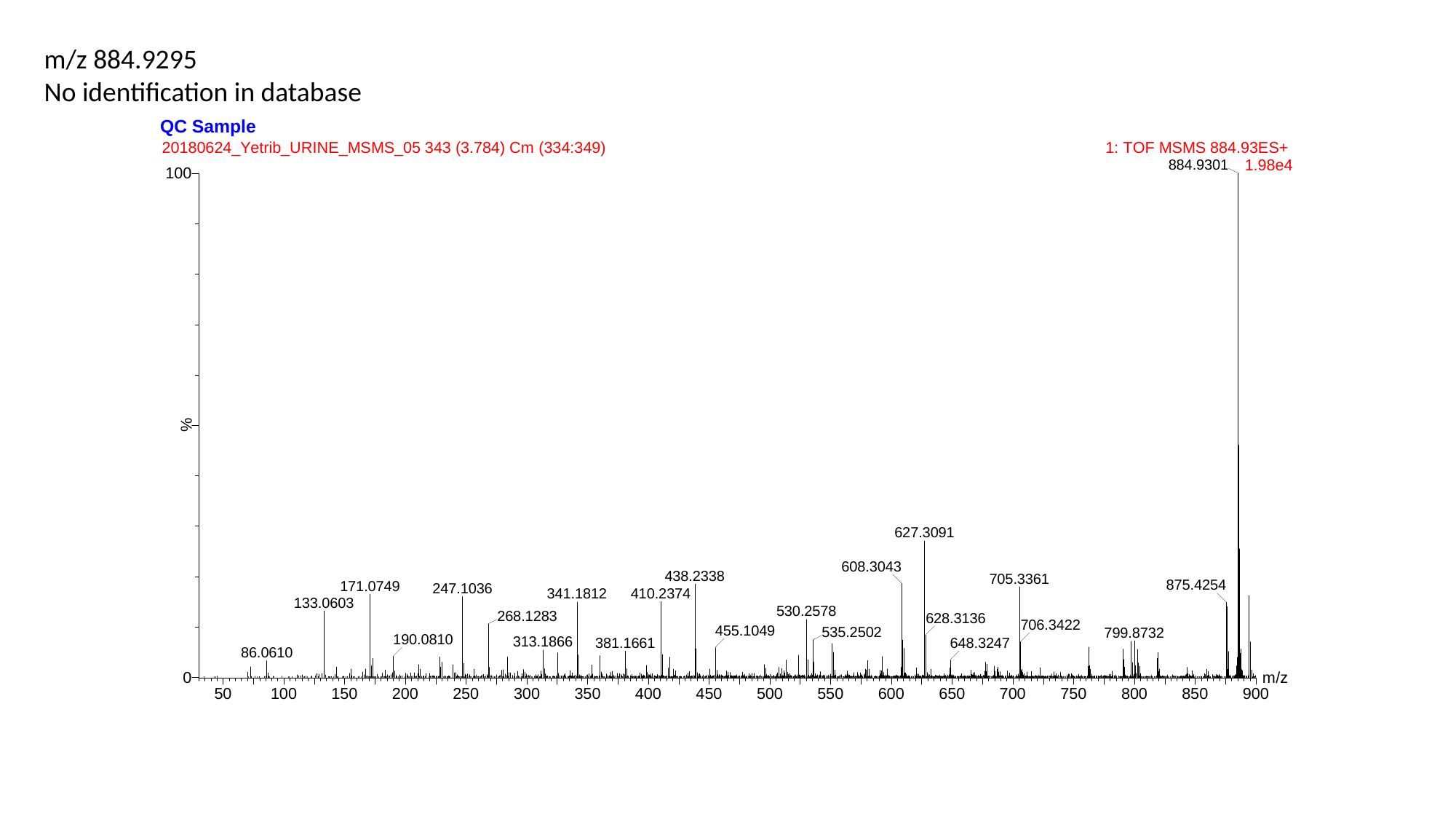

m/z 884.9295
No identification in database

## Slide 3
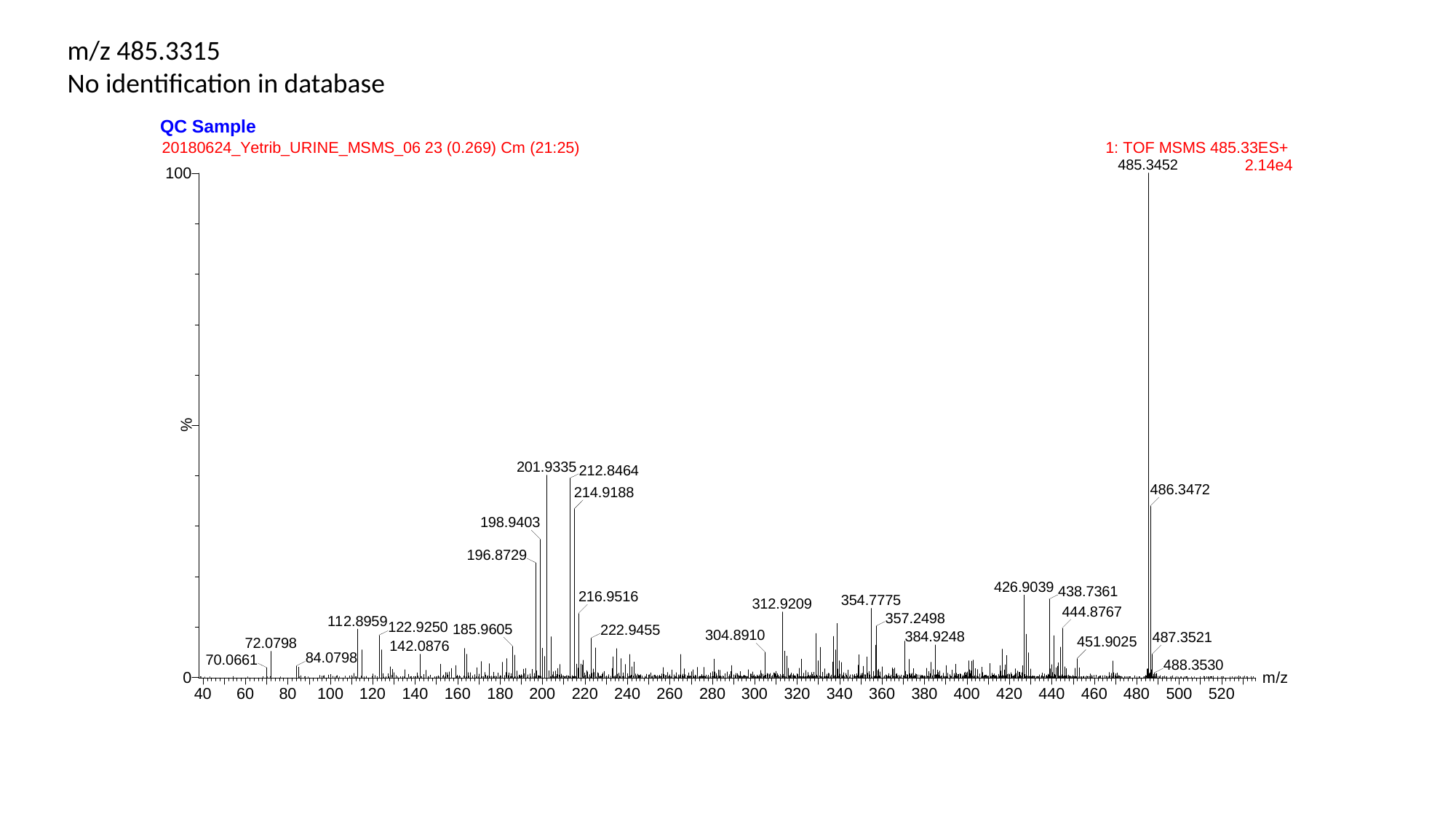

m/z 485.3315
No identification in database

## Slide 4
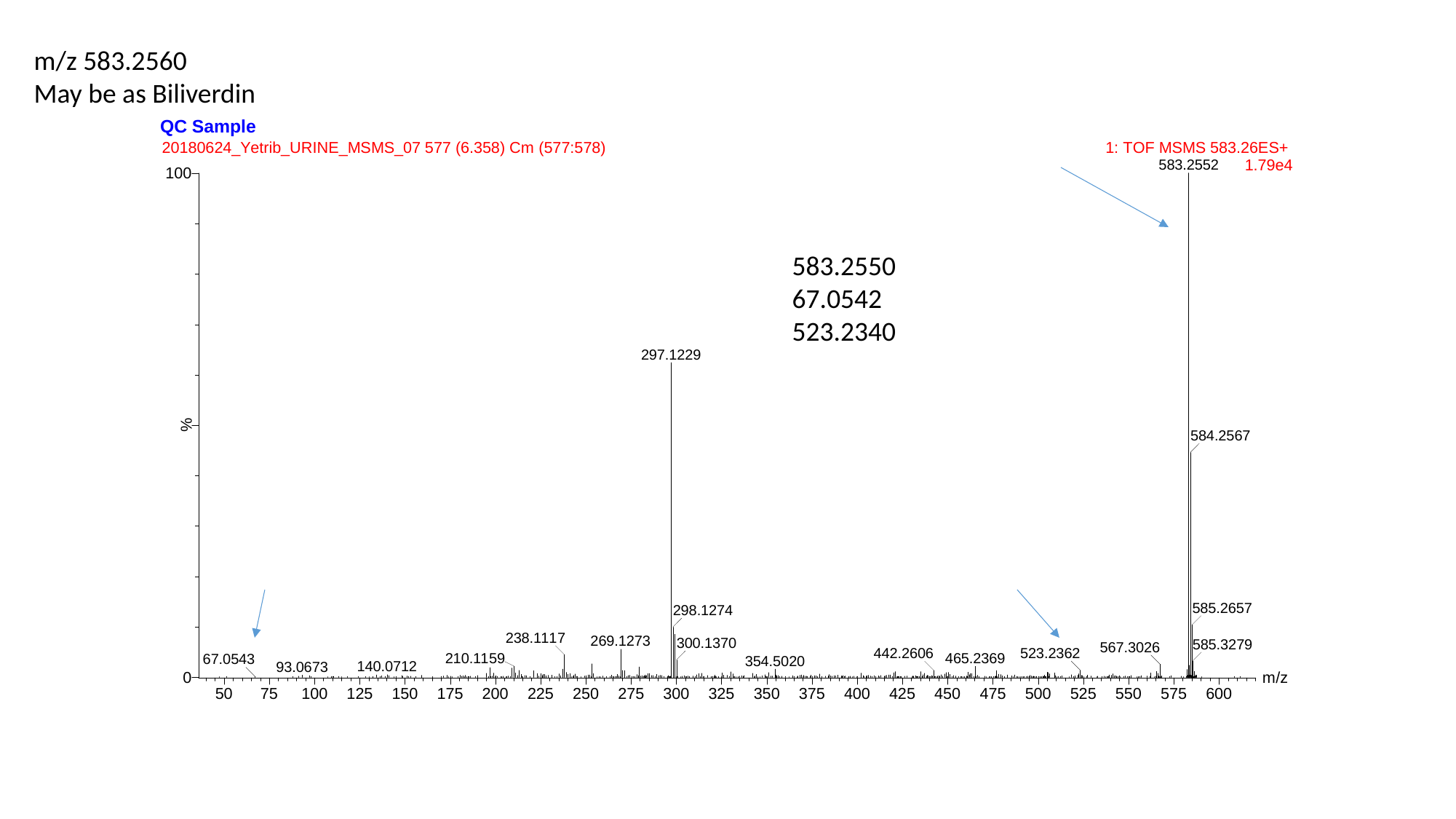

m/z 583.2560
May be as Biliverdin
583.2550
67.0542
523.2340

## Slide 5
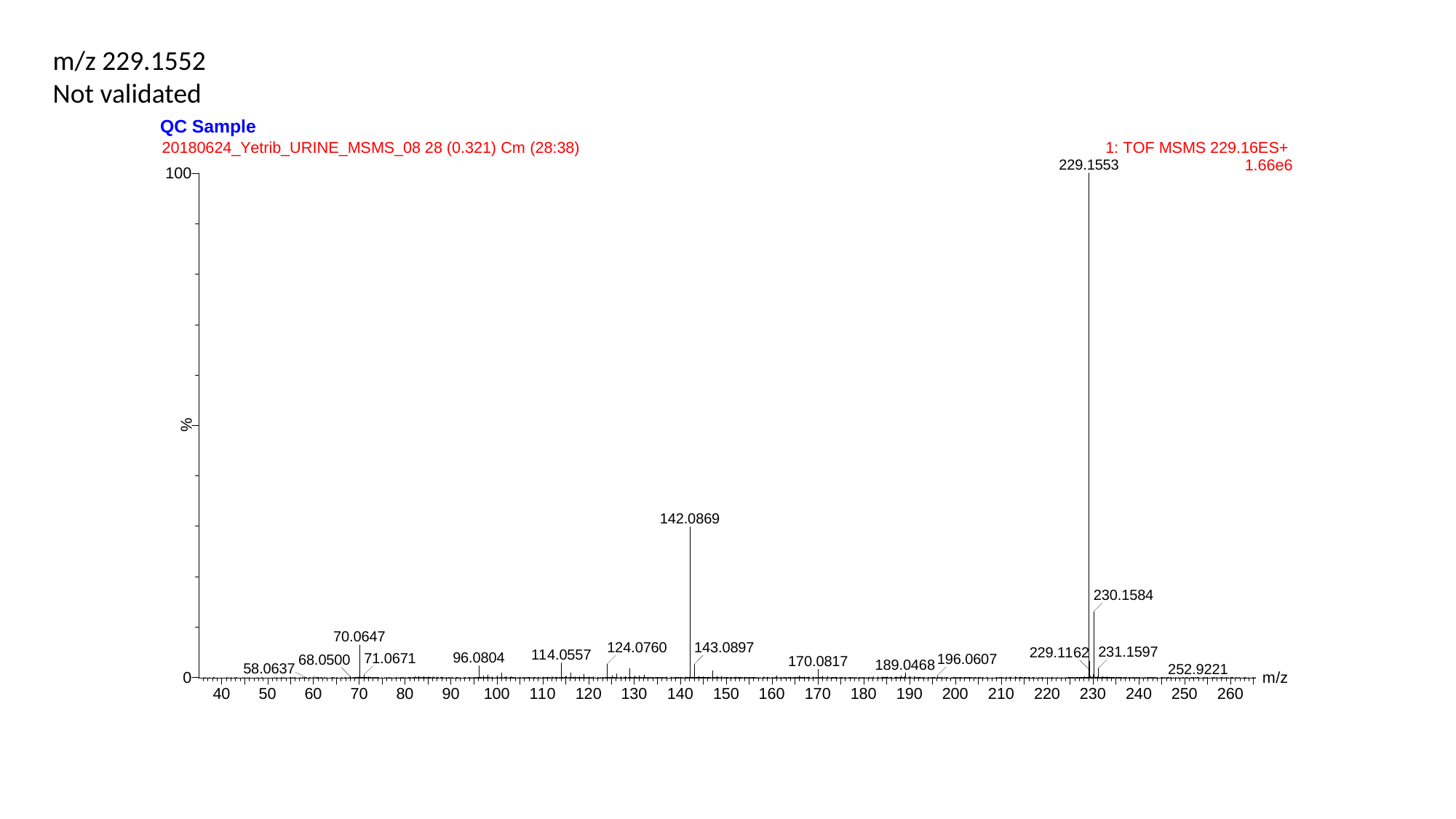

m/z 229.1552
Not validated

## Slide 6
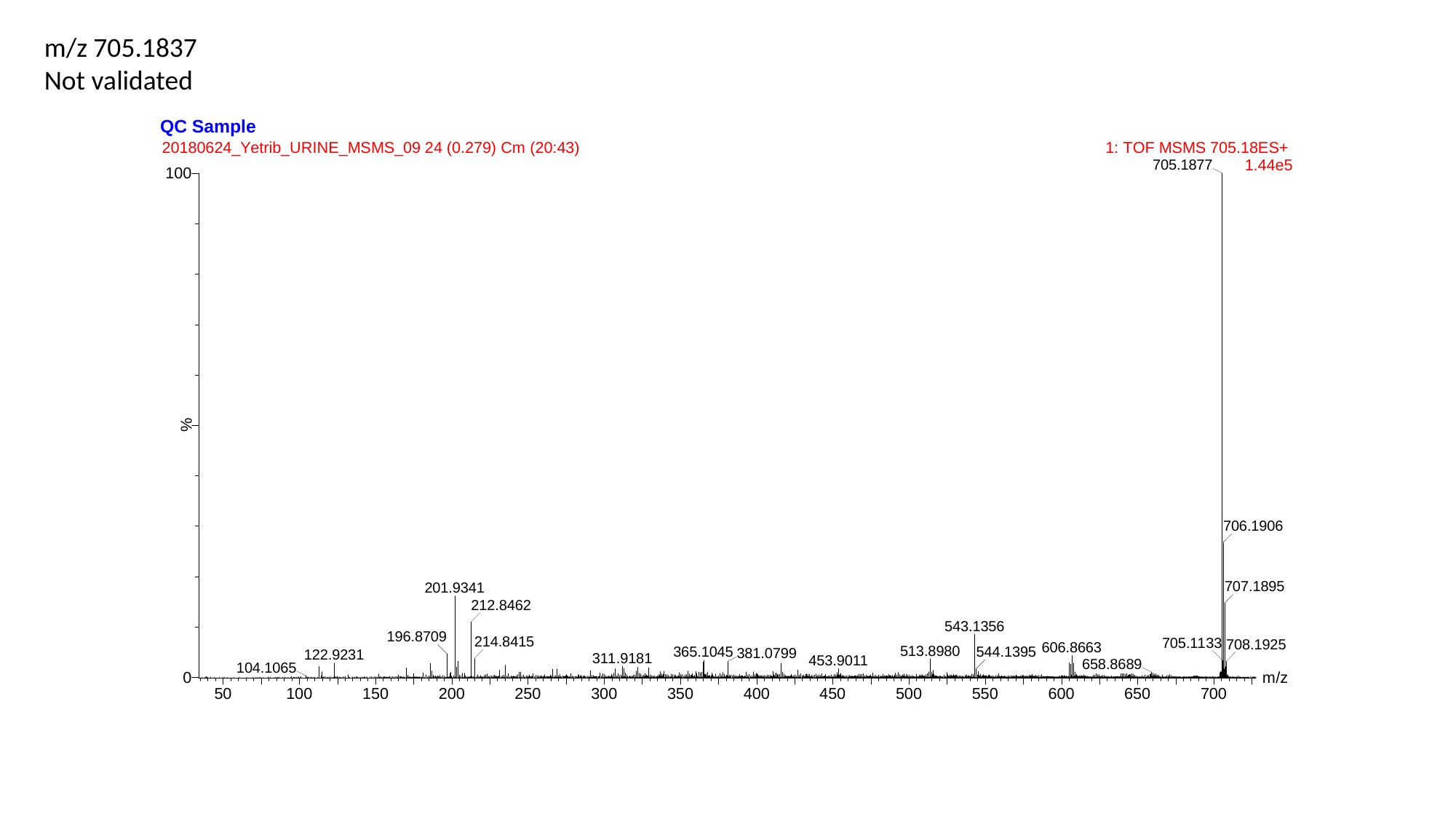

m/z 705.1837
Not validated

## Slide 7
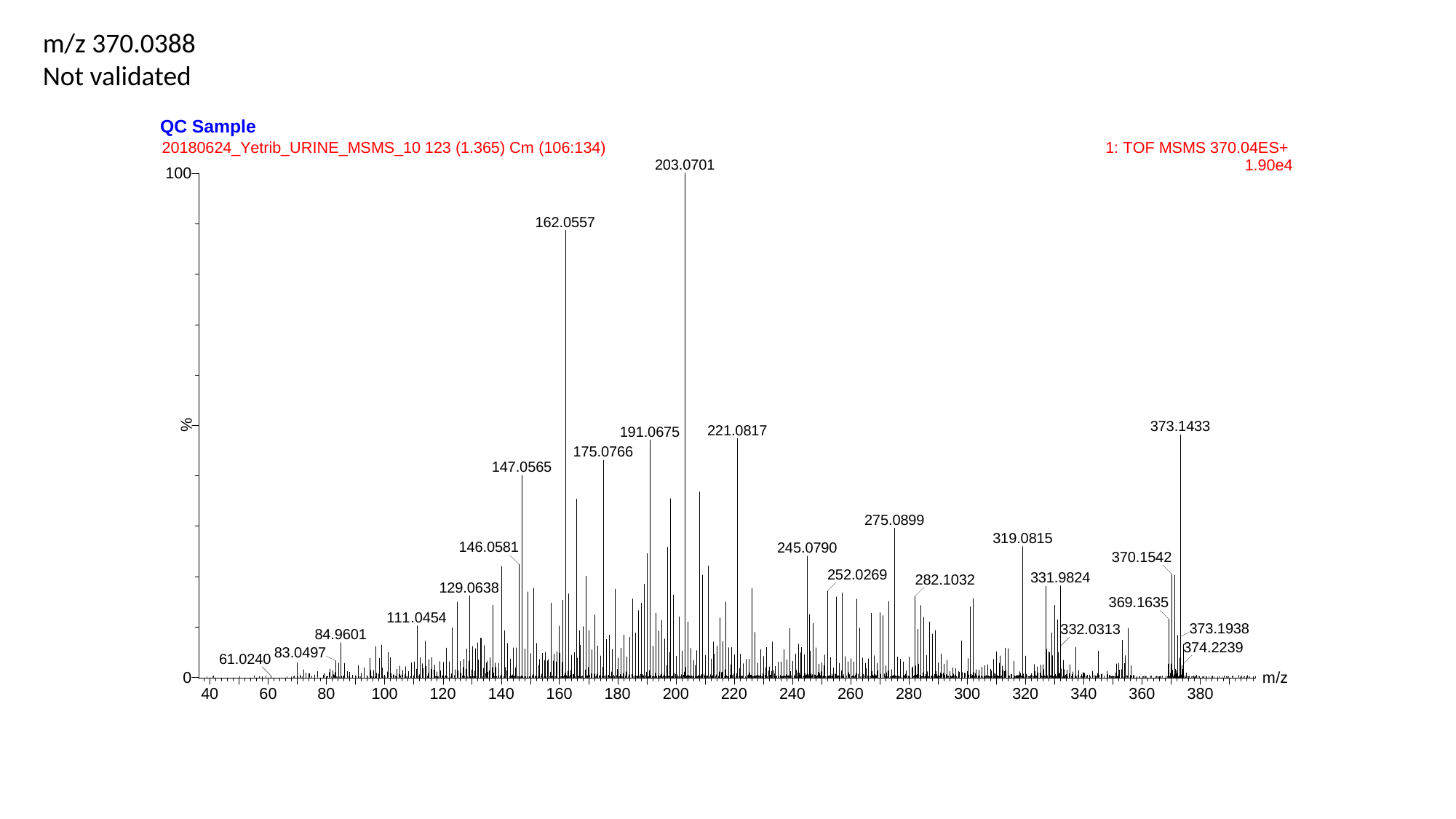

m/z 370.0388
Not validated

## Slide 8
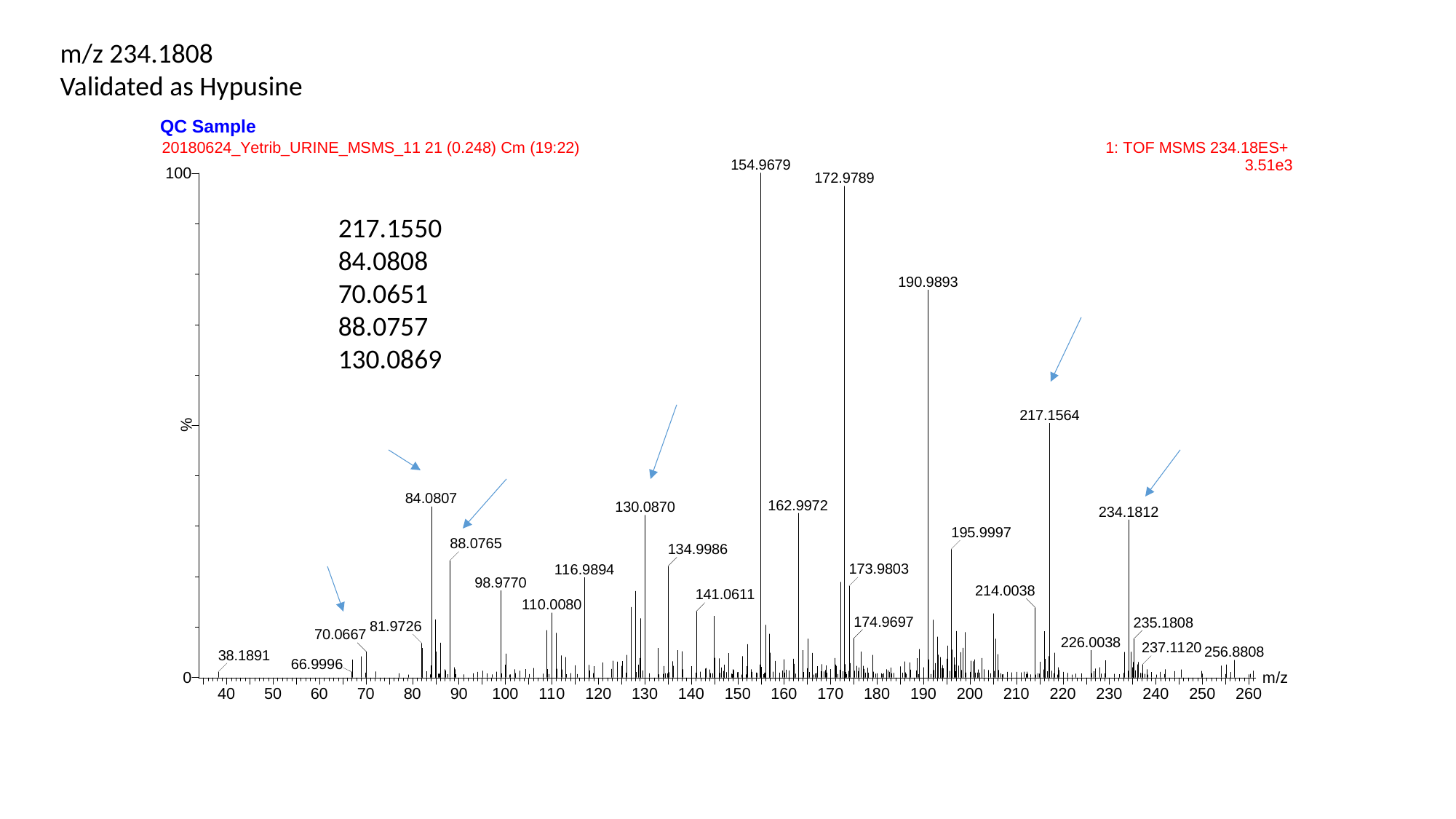

m/z 234.1808
Validated as Hypusine
217.1550
84.0808
70.0651
88.0757
130.0869

## Slide 9
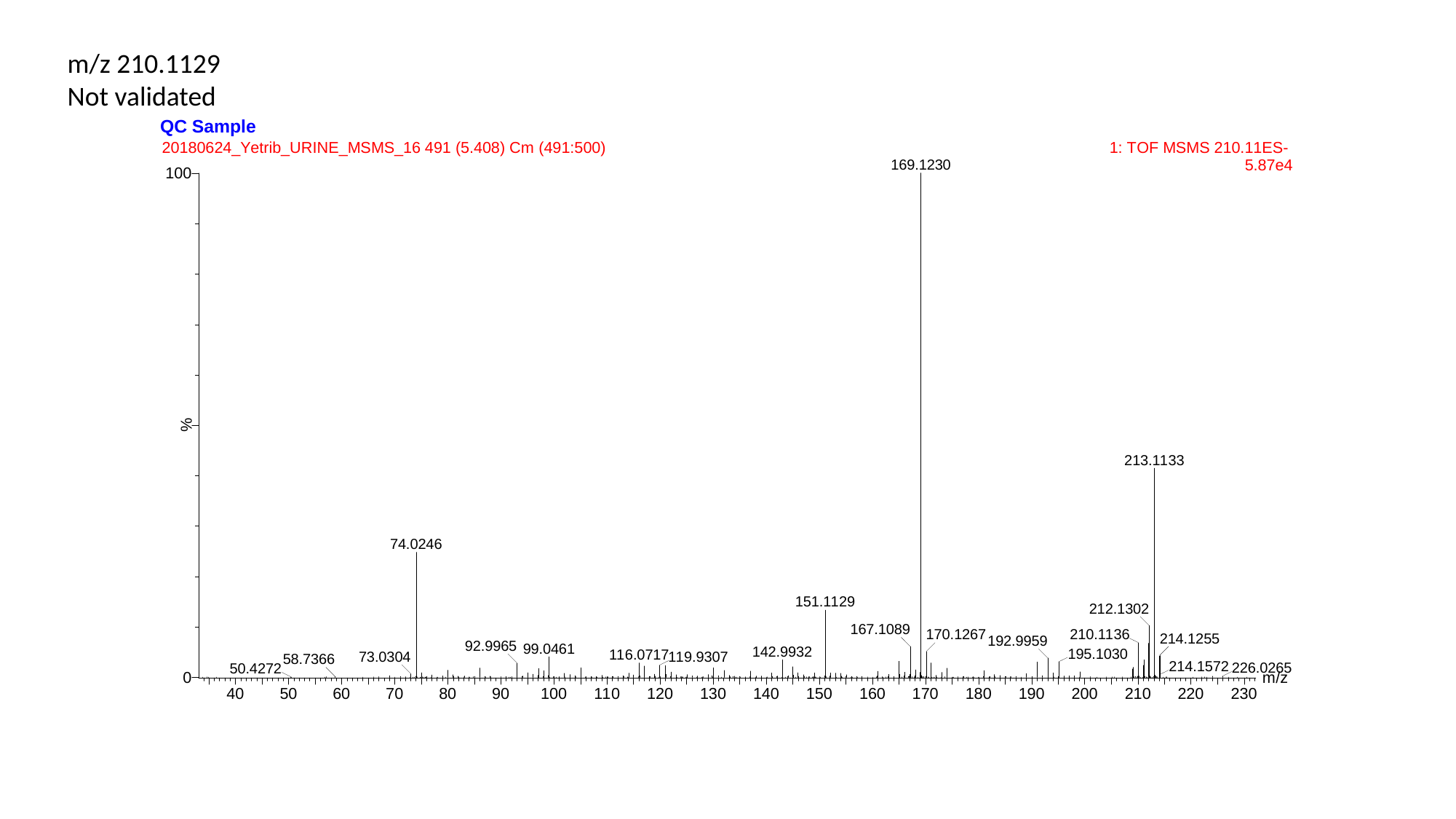

m/z 210.1129
Not validated

## Slide 10
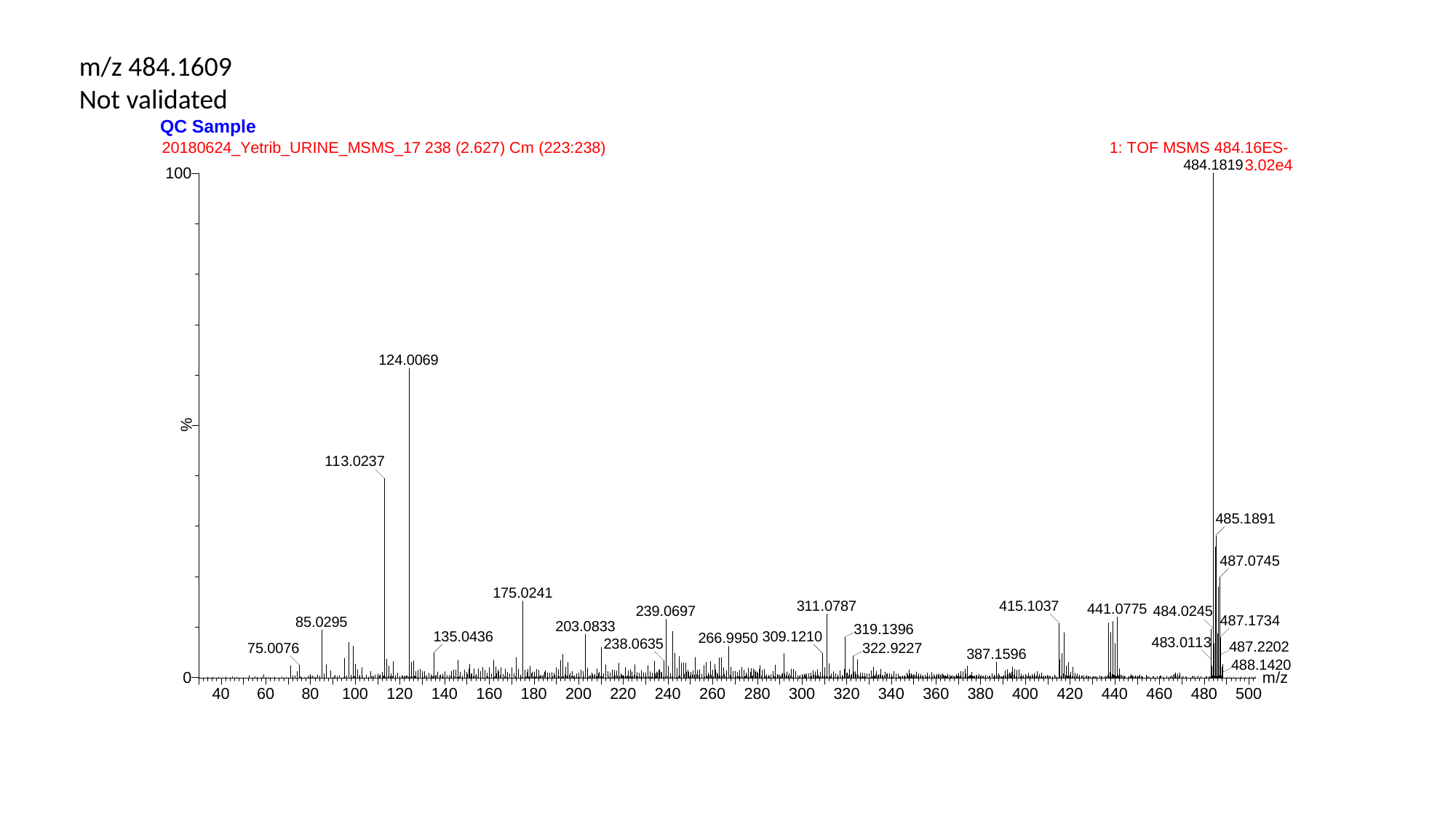

m/z 484.1609
Not validated

## Slide 11
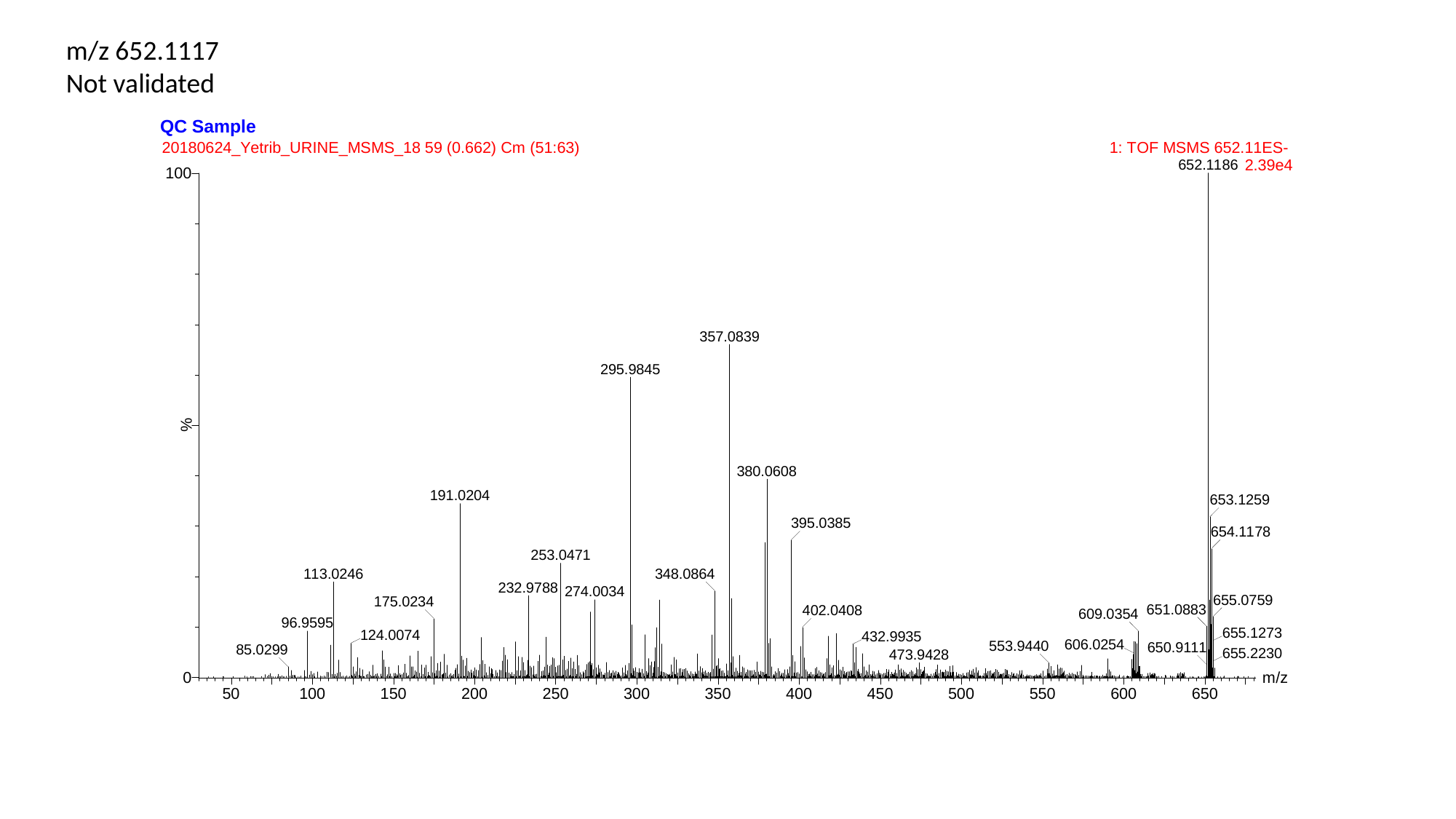

m/z 652.1117
Not validated
