# Supplementary figures and images for: Discovery of potential urine-accessible metabolite biomarkers associated with muscle disease and corticosteroid response in the mdx mouse model for Duchenne
Source: PLoS One. 2019 Jul 16;14(7):e0219507. doi: 10.1371/journal.pone.0219507 (PMC6634414; doi:10.1371/journal.pone.0219507)

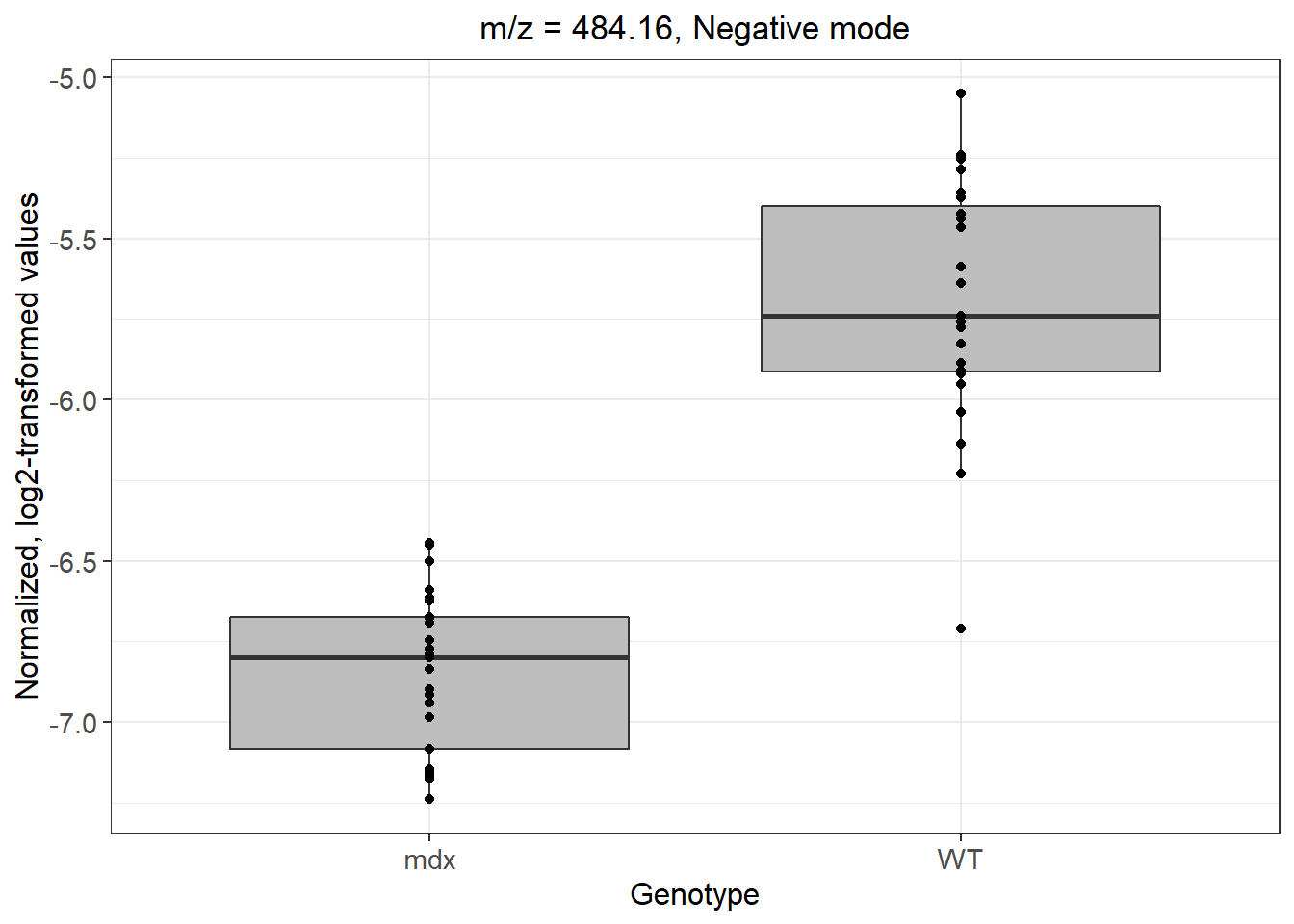

Supplement: S2 Fig — (TIF) [file pone.0219507.s002.tif]

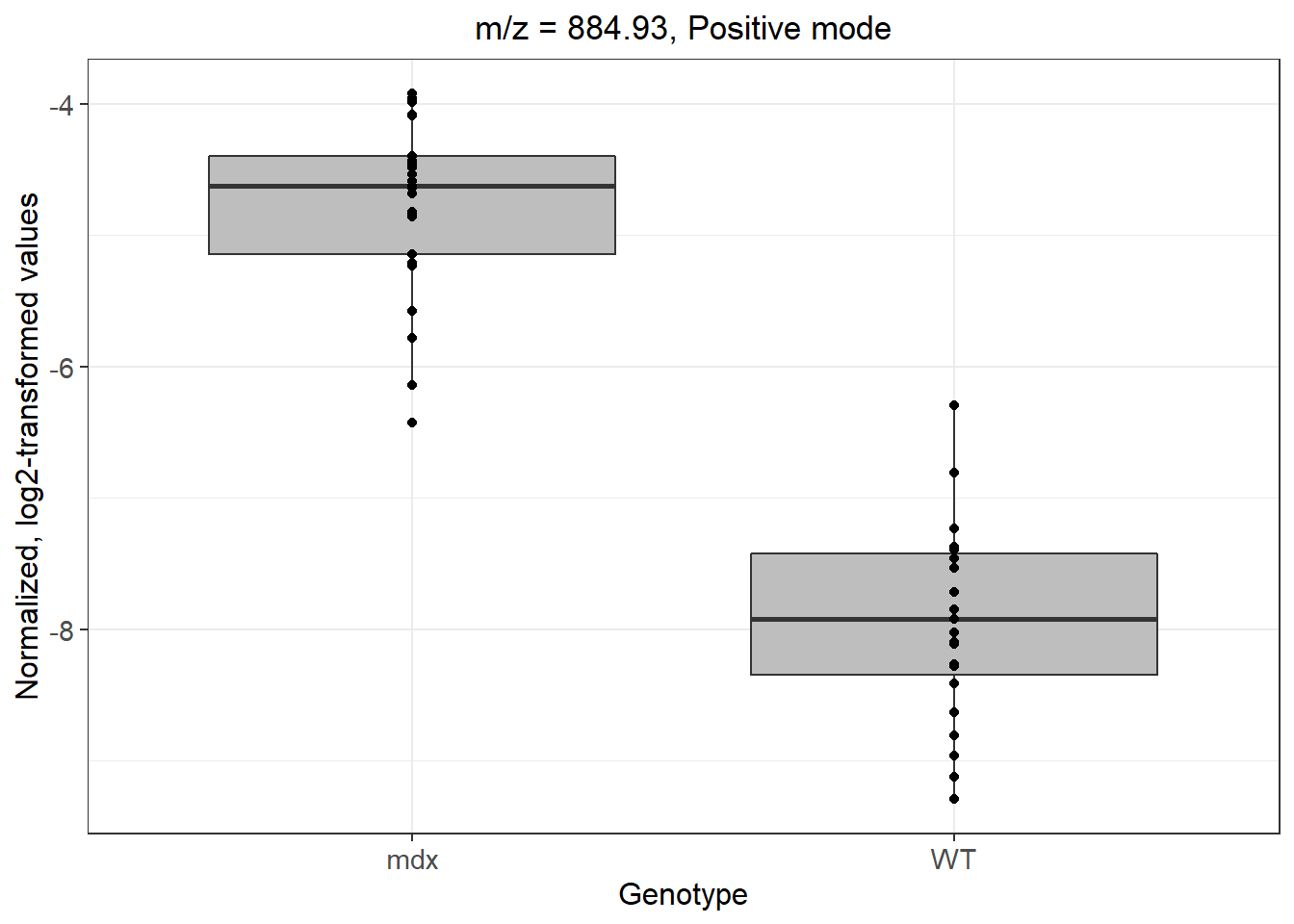

Supplement: S3 Fig — (TIF) [file pone.0219507.s003.tif]
